# Elucidating intrinsic contribution of *d*-orbital states to oxygen evolution electrocatalysis in oxides

**DOI:** 10.1038/s41467-021-21055-0

**Published:** 2021-02-05

**Authors:** Tae Gyu Yun, Yoon Heo, Hyung Bin Bae, Sung-Yoon Chung

**Affiliations:** 1 Department of Materials Science and Engineering, and KAIST Institute for the Nanocentury, Korea Advanced Institute of Science and Technology, 291 Daehak-ro, Yuseong-gu Daejeon, 34141 Korea; 2 KAIST Analysis Center, Korea Advanced Institute of Science and Technology, 291 Daehak-ro, Yuseong-gu Daejeon, 34141 Korea

**Keywords:** Catalysis, Electron transfer, Materials for energy and catalysis, Surfaces, interfaces and thin films

## Abstract

Although numerous studies on oxide catalysts for an efficient oxygen evolution reaction have been carried out to compare their catalytic performance and suggest new compositions, two significant constraints have been overlooked. One is the difference in electronic conduction behavior between catalysts (metallic versus insulating) and the other is the strong crystallographic surface orientation dependence of the catalysis in a crystal. Consequently, unless a comprehensive comparison of the oxygen-evolution catalytic activity between samples is made on a crystallographically identical surface with sufficient electron conduction, misleading interpretations on the catalytic performance and mechanism may be unavoidable. To overcome these limitations, we utilize both metallic (001) LaNiO_3_ epitaxial thin films together with metal dopants and semiconducting (001) LaCoO_3_ epitaxial thin films supported with a conductive interlayer. We identify that Fe, Cr, and Al are beneficial to enhance the catalysis in LaNiO_3_ although their perovskite counterparts, LaFeO_3_, LaCrO_3_, and LaAlO_3_, with a large bandgap are inactive. Furthermore, semiconducting LaCoO_3_ is found to have more than one order higher activity than metallic LaNiO_3_, in contrast to previous reports. Showing the importance of facilitating electron conduction, our work highlights the impact of the near-Fermi-level *d*-orbital states on the oxygen-evolution catalysis performance in perovskite oxides.

## Abstract (web summary)

Despite many studies on the oxygen-evolution perovskite-type electrocatalysts thus far, significant constraints prevent precise evaluations of catalytic activity. Here authors examine over 50 heteroepitaxial LaNiO_3_ and LaCoO_3_ (001) thin films to assess the intrinsic catalytic behaviors.

## Introduction

As many devices for electrochemical energy storage and conversion operate at room temperature, control of electrochemical redox reactions is of great significance to reduce the activation barriers and thereby boost the overall storage and conversion efficiencies. Among different types of redox reactions, the oxygen evolution reaction (OER) is an indispensable kinetic process taking place at the anode during water splitting in electrolyzers and at the cathode during charging in metal–air rechargeable batteries^1–3^. In particular, multiple transfers of electrons and protons during the OER are considered to result in a much large activation barrier^4^, compared with that of the hydrogen evolution reaction. The utilization of efficient OER electrocatalysts is thus imperative to significantly reduce the overpotential of the anodic reaction in water electrolysis for hydrogen production.

In addition to traditional Ir- and Ru-based oxides^5–8^, many other oxide materials have been suggested as promising OER catalysts over the last decade^9,10^, encompassing complex perovskite oxides^11–16^, layered (oxy)hydroxides^17–20^, and spinel oxides^21,22^. Furthermore, as several notable descriptors have been successfully developed with the aid of ab initio density functional theory (DFT) calculations^4,23^, they enable us to understand the overall origin of activity variations and sometimes even to theoretically predict the catalytic properties when new catalysts are reported. As known well, charge transfer between adsorbates (O* and OH*) and a metal (M) on the catalyst surface will always be involved in the OER^4^. In this regard, electronic structure-associated descriptors recently have been proposed^24–33^ in addition to the early descriptor based on the bond strength between M and the adsorbates since the work by Bockris and Otagawa^11,12^. The filling of the *e*_g_-level electrons of the metal cations^24^, the position of the O *p*-band center^25^, the degree of covalency between transition metals and oxygen^26,30^, and the charge-transfer energy^13,29^ are noticeable examples of the important descriptors on the basis of the electronic structure of oxides.

Although these recent descriptors provide reasonable insight and further prediction in determining the relative OER activity between metal oxides, serious constrains should be noted when the experimentally obtained activities are compared with the theoretical descriptors. First, if a catalyst is electronically insulating, a great deal of applied overpotential may be dissipated as Ohmic resistance, resulting in much lower OER current even though the catalyst may be intrinsically very active. Therefore, a direct comparison of the OER activity cannot be made when one catalyst is metallic and the other is insulating or semiconducting with a bandgap. Second, as demonstrated in several oxides including SrRuO_3_^34,35^, the OER activity of crystalline catalysts is considerably orientation-dependent, showing fairly different values of the OER current density on each crystalline facet. This indicates that the precise difference of the OER activity between catalysts is difficult to identify unless an identical crystallographic surface is measured. Third, most theoretical studies regarding the variation of M−O(H)* bond strength have dealt with merely one specific facet^4,23^, despite that the experimentally measured OER properties come from numerous random facets of polycrystalline catalysts. Moreover, many DFT calculations to obtain the density of states (DOS) of M *d* and O *p* orbitals have been carried out by using the bulk supercells rather than the supercells containing the surface.

To overcome these limitations in this work, we utilize heteroepitaxial (001)_cubic_ thin films of LaNiO_3_ and LaCoO_3_ perovskite oxides instead of polycrystalline particles^36–39^. In one series of experiments, metallic LaNiO_3_ thin films doped with a small amount of seven different trivalent metal dopants, Fe, Co, Cr, Mn, Sc, Al, and In are used. As these dopants are all 3+, neither cation vacancies nor oxygen vacancies are created during thin-film fabrication. As a result, we can straightforwardly focus on the correlation between the OER activity and the constitution from the transition-metal *d*-orbital states by doping the identical (001) metallic surface without interference by the charged point defects and the crystallographic orientation difference. One of the significant findings in this study is that Fe, Cr, and Al, the counter La-perovskites of which (LaFeO_3_, LaCrO_3_, and LaAlO_3_) are known to be OER inactive^11,12,14^, make a notable contribution to the OER activity when doped in metallic LaNiO_3_ films. The DFT calculations also consistently demonstrate the appearance of a large density of the *d*-orbital states near the Fermi level in cases where the OER activity is enhanced. In addition, when the low conductivity limitation is eliminated in semiconducting LaCoO_3_ films by introducing a metallic interlayer in the other series of experiments, the OER current density of the LaCoO_3_ film is observed to be one order higher than that of the LaNiO_3_ film, in stark contrast to previous results^11,12,14,40^, showing the best OER activity among La*M*O_3_-type perovskite oxides. In addition to clarifying the importance of the electronic conductivity of OER catalysts, our study dealing with more than 50 heteroepitaxial thin films of different compositions highlights the impact of the near-Fermi-level *d*-orbital states on the OER performance in perovskite oxides.

## Results

### DFT calculations for DOS

**Fig. 1:**
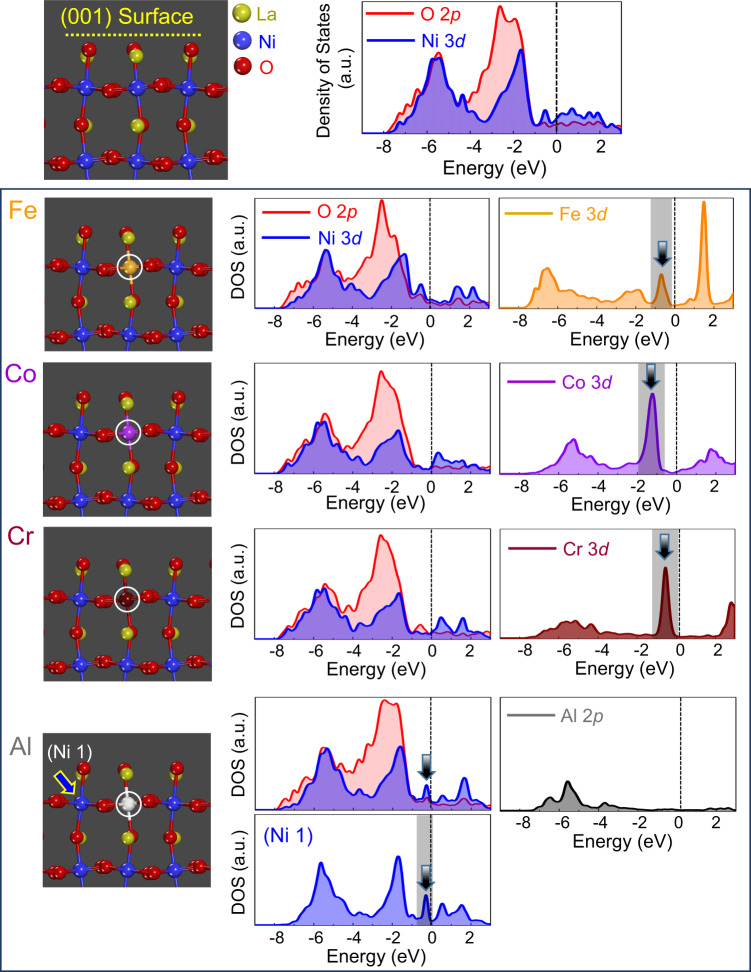
DOS at the LaNiO_3_ (001)_cubic_ surface with various dopants (1). The first set of DOS plots for O 2*p*, Ni 3*d*, and metal 2*p* or 3*d* states is provided for each case. A black arrow together with a gray shadow in each plot indicates the noticeably high density of the 3*d* states near the Fermi level. It is noteworthy that a rise of the 3*d* states of Ni-1 is induced by Al doping, although Al does not provide a significant contribution to the total DOS between −2 and 0 eV.

**Fig. 2:**
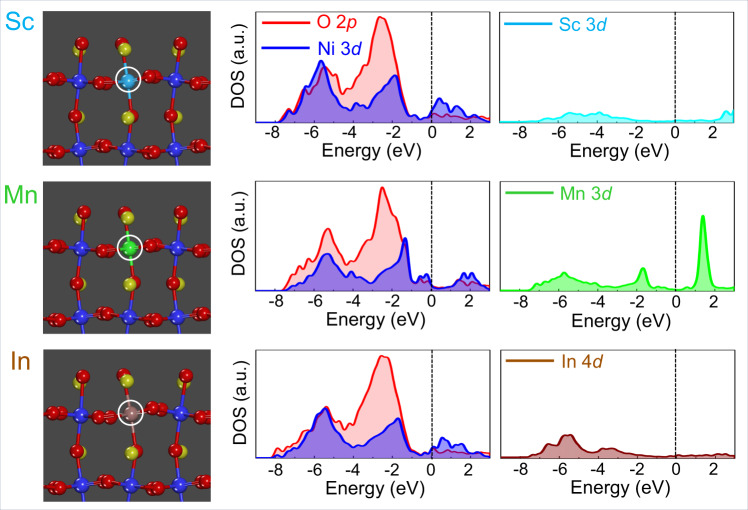
DOS at the LaNiO_3_ (001)_cubic_ surface with various dopants (2). The second set of DOS plots for O 2*p*, Ni 3*d*, and metal 3*d* states is provided for each case. In contrast to the first set for Fe, Co, Cr, and Al doping, the dopants, Sc, Mn, and In, have neither a substantial influence on the total DOS nor induce any Ni 3*d*-state variation near the Fermi level.

Prior to directly measuring the OER current density of each thin-film sample, we carried out DFT calculation to acquire the DOS information at the (001) LaNiO_3_ surface with dopants. A series of DOS plots of Ni 3*d*, O 2*p*, and dopant metal *p* and *d* orbitals shown in Figs. 1 and 2 were obtained from the first-row octahedra at the surface without La. A readily recognizable feature in Fig. 1 is that a significantly high density of the 3*d* states of three dopants, Fe, Co, and Cr (white circles in the schematic illustrations of the supercells), is commonly identified between −2 and 0 eV below the Fermi level, as denoted by a black arrow in each of the three plots. In contrast, the O 2*p* DOS in each plot does not substantially vary with doping when compared with that of the pristine (001) surface.

Another intriguing aspect can be found in the Al-doped surface. As indicated by a black arrow in the DOS of “Ni-1,” a noticeable increase of Ni 3*d* DOS between −1 and 0 eV below the Fermi level is identified in the plot, demonstrating a doping-induced increment of the Ni 3*d* states, whereas no major contribution of the Al 2*p* states independently to the total DOS is verified between −2 and 0 eV. This variation of the Ni 3*d* DOS induced by doping near the Fermi level is also observed in the Fe-doped surface (see Supplementary Fig. 1 for the DOS plots of the Fe-doped surface). By contrast, as shown in Fig. 2, neither significant density of the *d*-orbital states from the other three dopants, Sc, Mn, and In, below the Fermi level, nor notable variation in the Ni 3*d* states was obtained for the (001) surface. Based on the series of DOS plots provided in Figs. 1 and 2, two distinct types of trivalent dopants can be categorized (Fe, Co, Cr, and Al vs. Sc, Mn, and In), depending on how significantly the *d*-orbital states contribute to the total DOS near the Fermi level.

### Initial OER activities of doped LaNiO_3_ films

**Fig. 3:**
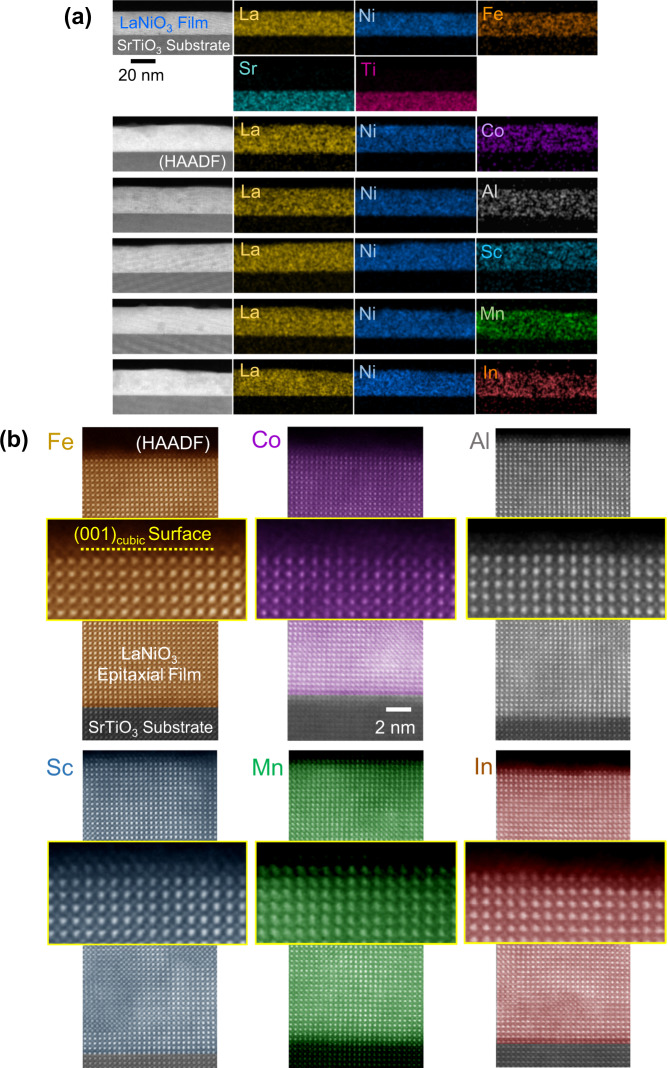
Fabrication of LaNiO_3_ heteroepitaxial (001)_cubic_ thin films with dopants. **a** A series of EDS maps is provided to verify the homogeneous distribution of the dopants in the films. The doping level was adjusted to be 10%. **b** HAADF images and their enlargements for the surface region are shown to directly confirm both the heteroepitaxy and the clean (001) surface of the films on SrTiO_3_ substrates.

To measure and compare the initial OER current values during the first anodic cycle from the identical crystallographic surface of doped LaNiO_3_, we fabricated heteroepitaxial (001) LaNiO_3_ thin films deposited on SrTiO_3_ single-crystal substrates by using a sol–gel method^36,41^. The heteroepitaxy nature of the films was first verified by X-ray diffraction selectively showing the (00 *l*) Brag reflections (see Supplementary Fig. 2 for the detailed X-ray patterns). Based on this X-ray diffractometry, both high crystallinity and heteroepitaxy of the films appear to be preserved up to 20% doping. As demonstrated in a series of composition maps acquired by energy-dispersive X-ray spectroscopy (EDS) along with high-angle annular dark-field (HAADF) scanning transmission electron microscopy (STEM) images in Fig. 3a, each of the dopants was also verified to be homogeneously distributed in a film with 25–30 nm thickness. In addition, we verified the trivalent state of dopants by using X-ray photoemission spectroscopy (XPS) and electron energy-loss spectroscopy (EELS) (see Supplementary Figs. 3 and 4 for details)

As the Cr-*K*_α1_ peak (5.42 keV) in EDS overlaps with the La-*L*_β2_ peak (5.38 keV), the EDS analysis cannot be applied to La-containing materials when a compositional analysis of Cr is necessary (see Supplementary Fig. 5 for more details on the artifact of Cr detection in EDS). We thus used EELS in STEM instead to investigate the Cr distribution. In contrast to the successful fabrication of doped LaNiO_3_ epitaxial thin films for six dopants, homogeneous incorporation of Cr was found to be very difficult, as excessively rapid gelation of the Cr precursor was unavoidable during the sol–gel process. Consequently, as shown in Supplementary Fig. 6, serious inhomogeneity of Cr in the film and the formation of secondary phases on the film surface were frequently observed in Cr-added films during the STEM and EELS observation. Atomic-column-resolved cross-sectional HAADF-STEM images provided in Fig. 3b directly confirm the high crystallinity of each film with the perovskite structure and a clean and flat (001) surface without secondary impurity phases for the six dopants except the Cr case. Supplementary Fig. 7 shows the (001) surface of pristine LaNiO_3_ films without impurity or amorphous phases as well.

**Fig. 4:**
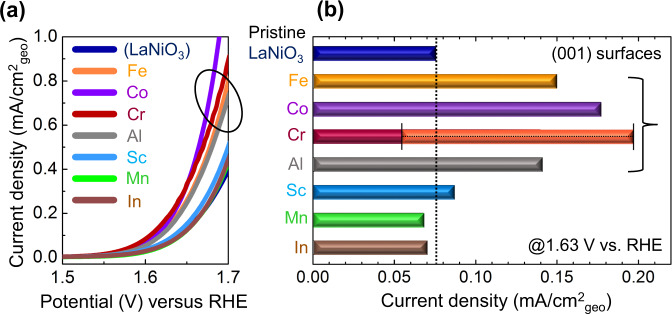
OER activity comparison of the doped LaNiO_3_ (001)_cubic_ surfaces. **a** This plot shows the variation of the OER current density of the films with 5% doping as a function of applied potential vs. RHE. **b** The bar graph compares the difference between the OER current densities of the doped (001) surfaces measured at 1.63 V vs. RHE. For an easier comparison, a black vertical line is added on the graph, showing the OER current density of the pristine (001) surface. As denoted, Fe, Co, Cr, and Al have a notably beneficial effect on the OER activity, while Sc, Mn, and In make little contribution.

The optimum doping concentration in each film sample for the OER activity measurement was adjusted to be comparatively small, 5%, as the metallic conduction behavior of the matrix LaNiO_3_ should be maintained so as to have sufficiently high electronic conductivity on an order of 10^3^ S/cm. Figure 4a presents the OER current densities of the (001) surfaces during the forward sweep of the first cycle as a function of the anodic potential vs. reversible hydrogen electrode (RHE). The OER activity of our pristine (001) LaNiO_3_ films in this work (~75 μA/cm^2^) is exactly comparable with the activity (30–90 μA/cm^2^) of (001) LaNiO_3_ films reported in previous studies^42–44^ (see Supplementary Fig. 8 for details). As specifically represented in the bar graph in Fig. 4b, the catalytic activity of Fe-, Co-, Cr-, and Al-doped (001) films shows more than twofold enhancement, compared with that of the pristine film. This improvement by a small amount of doping is thus a significant indication that Fe, Cr, and Al in addition to Co can be beneficial for the OER catalysis if sufficiently high electronic conductance is supported. Although 6 out of 11 Cr-doped thin-film samples exhibit remarkable OER activity enhancement, no improvement is observed in the other five samples (see Supplementary Fig. 9 for details). We therefore denote this variation by using a different color in the bar graph for Cr along with black lines in Fig. 4b to indicate the maximum and minimum values. This wide range of variation in the catalytic activity among Cr-doped samples appears to be attributable to the serious inhomogeneity of Cr, as shown in Supplementary Fig. 6. Nevertheless, the observation of enhanced activity by Cr demonstrates that Cr is a catalytically active dopant in LaNiO_3_.

In contrast to these four dopants, Sc, Mn, and In were identified to have little influence on the OER catalysis, revealing nearly the same OER current density with that of the pristine film, as shown in Fig. 4b. Entire sets of the OER current values and the Tafel slopes with different doping levels (up to 20%) for each of the dopants are provided in Supplementary Fig. 10 and Supplementary Table 1. As all the thin-film samples were fabricated in an identical manner, the double-layer (DL) capacitance representing the electrochemically active surface area^9,45,46^ does not vary between the samples regardless of the doping species (see Supplementary Fig. 11 and Supplementary Table 1 for the DL capacitance values). Furthermore, as demonstrated in Supplementary Fig. 12, the nearly invariable surface topologies obtained by atomic force microscopy (AFM) and the merely 15% variation of the DL capacitances after OER cycling confirm that the notable enhancement of the OER activities by addition of Fe, Co, Cr, and Al shown in Fig. 4 stems from the doping effect rather than surface morphology variation. An XPS analysis also verifies no serious dopant dissolution during the anodic cycling (see Supplementary Fig. 13 for the XPS results).

A combination of the series of DOS plots shown in Figs. 1 and 2, and the OER activity results in Fig. 4 reasonably suggests a noticeable correlation between the *d*-orbital states and the OER electrocatalysis. As indicated by black arrows in Fig. 1, the substantially high density of 3*d* states between −2 and 0 eV below the Fermi level is a common feature of Fe, Co, and Cr dopants for the significantly improved OER catalytic activity. Even though there is no contribution of Al to the DOS neat the Fermi level, it is noted that Al doping induces a rise of the 3*d* DOS of neighboring Ni, as also denoted by a black arrow in Fig. 1. In particular, the results of OER enhancements by Cr and Al are notable new findings because their counterpart perovskites, LaCrO_3_ and LaAlO_3_, are known to be inactive to the OER catalysis.

### OER activities of La(Ni,Co)O_3_ solid-solution films

**Fig. 5:**
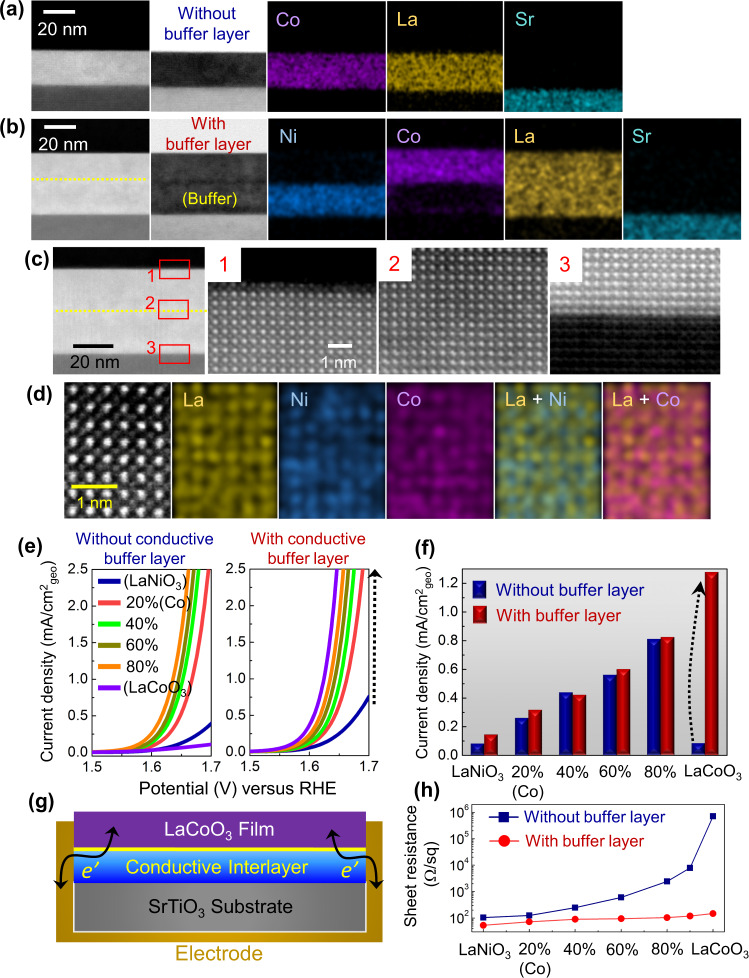
La(Ni,Co)O_3_ (001) solid-solution thin films. Two different configurations of thin-film samples with and without a conductive interlayer were prepared. **a**, **b** Two sets of EDS maps along with a pair of HAADF- and BF-STEM images exemplify LaCoO_3_ films (**a**) directly deposited on a SrTiO_3_ substrate and (**b**) with a LaNiO_3_ conductive interlayer on a substrate. **c** Atomic-scale HAADF images verify the heteroepitaxy among the film, the interlayer, and the substrate, showing the preservation of their perovskite structure. **d** This set of atomic-scale EDS maps shows homogeneous distribution of Ni and Co in a La(Ni_0.4_Co_0.6_)O_3_ film. **e** The current–potential variations of La(Ni,Co)O_3_ solid-solution thin films with and without a conductive interlayer are plotted. **f** This bar graph represents the OER current densities measured at 1.63 V vs. RHE from the plots in **e** for comparison. In addition to consistent increment of the OER current density with Co addition up to 80%, a huge difference of the OER activity between LaCoO_3_ films with and without a conductive interlayer is noted. **g** A schematic illustration indicates that the conductive interlayer provides a viable conduction path at the entire bottom surface of the film. **h** The sheet resistance was also measured in a series of La(Ni,Co)O_3_ solid-solution films. The conductive interlayer is verified to play a crucial role to efficiently reduce the sheet resistance of Co-rich films including the pristine LaCoO_3_ film.

To further examine the effect of doping and subsequent *d*-orbital states on the OER activity, we carried out another set of experiments using La(Ni,Co)O_3_ solid-solution thin films. As LaCoO_3_ has the same crystal structure (trigonal, space group: *R*$\bar 3$*m*) and very similar lattice parameters with LaNiO_3_, full-range solid solutions between LaCoO_3_ and LaNiO_3_ are achievable. However, it is noted that LaCoO_3_ is not metallic but semiconducting with a comparatively small bandgap (0.5–0.6 eV)^47^. Therefore, two different sample configurations were adopted for comparison. As shown in the STEM images and EDS maps in Fig. 5a, b, one configuration is a simple La(Ni,Co)O_3_ thin film directly deposited on a SrTiO_3_ substrate (Fig. 5a) and the other contains a conductive perovskite interlayer between the film and the substrate (Fig. 5b) to provide sufficient contact for electron conduction to the entire bottom surface of the film (see the schematic illustration in Fig. 5g). Metallic LaNiO_3_ was used for the conductive interlayer. The preservation of the epitaxy among the substrate, the interlayer, and the film was directly confirmed by atomic-scale STEM observation as shown in Fig. 5c. A whole cross-sectional HAADF image showing the entire configuration with the epitaxial interlayer at an atomic level is provided in Supplementary Fig. 14.

The solid-solution behavior between LaCoO_3_ and LaNiO_3_ was verified by observing the consistent shift of the (002) Brag reflection in thin-film samples during the X-ray diffractometry (see Supplementary Fig. 15 for a series of X-ray diffraction patterns of La(Ni_1−*x*_Co_*x*_)O_3_ (*x* = 0–1) films deposited on SrTiO_3_ substrates). Atomic-scale EDS maps^48–50^ in Fig. 5d specifically exemplify the homogeneous distribution of Ni and Co in a La(Ni_0.4_Co_0.6_)O_3_ film, directly proving the solid-solution mixture. When we measured the OER current density of a series of the La(Ni,Co)O_3_ solid-solution (001) films, an important finding could be readily acquired. As shown in Fig. 5e, f, a systematic enhancement of the OER activity with increasing Co substitution is identified for the samples with a metallic interlayer, demonstrating more than 10-fold higher OER current density of the (001) LaCoO_3_ surface (1.276 mA/cm^2^_geo_) than that of the (001) LaNiO_3_ surface (0.075 mA/cm^2^_geo_). It is worth noting that this remarkably large activity difference between LaCoO_3_ and LaNiO_3_ has not been reported thus far and previous studies rather have shown that LaNiO_3_ has the highest OER activity among many La-based perovskite catalysts^11,12,14^ (see Supplementary Fig. 16 for the comparison of the previously reported OER current densities of perovskite oxides). The considerably low OER current density of the (001) LaCoO_3_ surface without the conductive interlayer, 0.05 mA/cm^2^_geo_, which is even lower than that of the (001) LaNiO_3_ surface, straightforwardly reveals that exceptional OER activity of Co can be seriously interrupted if sufficient electron conduction to LaCoO_3_ is prevented. Indeed, the plot of sheet resistance of films in Fig. 5h consistently shows a reduction of resistance by four orders of magnitude in the LaCoO_3_ thin-film sample containing the interlayer. All the information regarding the OER current densities, the Tafel slopes, and the DL capacitance of La(Ni_1−*x*_Co_*x*_)O_3_ films with and without the conductive interlayer is provided in Supplementary Tables 2 and 3. In addition, the X-ray reciprocal space maps (RSMs)^51^ of the LaCoO_3_ thin film with the interlayer (the total thickness of >50 nm) demonstrate no substantial epitaxial strain (see Supplementary Fig. 17 for the RSM). As a consequence, the remarkably high OER activity observed in the LaCoO_3_ film with a LaNiO_3_ interlayer does not relate to the epitaxial strain.

### OER activities of doped LaCoO_3_ films

**Fig. 6:**
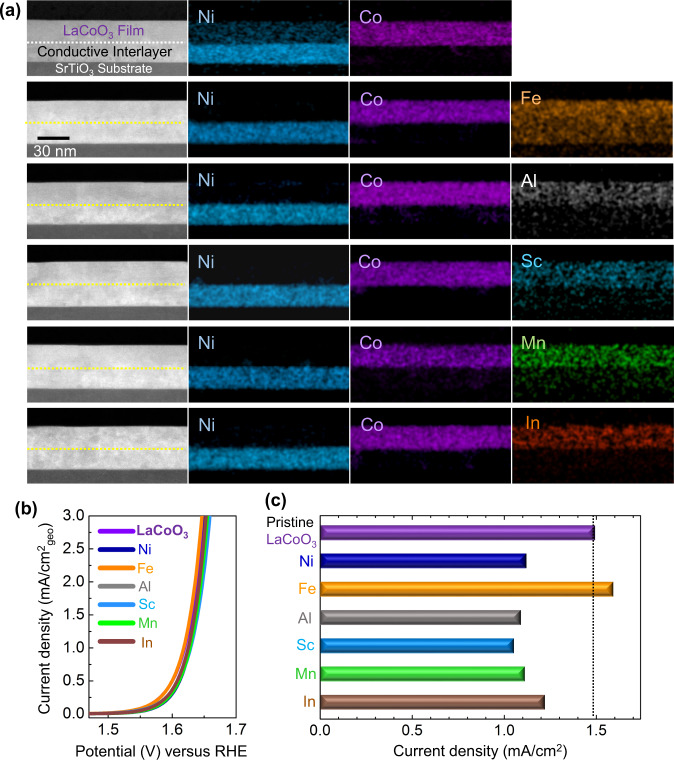
Fabrication of LaCoO_3_ heteroepitaxial (001)_cubic_ thin films with dopants. **a** A LaNiO_3_ metallic interlayer was utilized in all the thin films, as shown in the Ni map. This whole series of EDS maps also verifies the homogeneous distribution of the dopants in the films. The doping level was adjusted to be 10%. **b** This plot shows the variation of the OER current density of the films with 5% doping as a function of applied potential vs. RHE. No substantial enhancement of the OER current by doing is identified. **c** The bar graph indicates the OER current densities of the doped (001) surfaces measured at 1.63 V vs. RHE.

Our work shows that the intrinsic OER catalytic capability of LaCoO_3_ is exceptionally high, based on the results shown in Figs. 4 and 5 along with the prominently high density of Co 3*d* states near the Fermi level (see Supplementary Fig. 18 for the DOS of O 2*p* and Co 3*d* at the (001)_cubic_ surface in LaCoO_3_). In particular, if the conditions of a sufficient conduction path and nanosize catalyst morphology are satisfied to prevent serious Ohmic losses caused by the nonmetallic behavior of LaCoO_3_, the additional contribution of the 3*d* states from a small amount of dopants is anticipated to be marginal in contrast to the LaNiO_3_ cases. For the third set of experiments, we thus prepared (001) doped LaCoO_3_ thin-film samples with a conductive interlayer in an identical manner as in La(Ni,Co)O_3_ solid-solution (001) films. Figure 6a shows a series of EDS maps visualizing the homogeneous distribution of dopants in (001) LaCoO_3_ films with a metallic LaNiO_3_ interlayer on SrTiO_3_ substrates (see Supplementary Fig. 19 for the X-ray diffraction patterns of the films to prove the epitaxial growth). As compared in Fig. 6b, c, the dopant addition does not make a substantial contribution to the OER activity in LaCoO_3_, showing rather a detrimental influence in most cases of dopants, except for Fe. The electrochemical data including the OER current densities of doped LaCoO_3_ films with a conductive interlayer up to a 20% doping level are provided in Supplementary Table 4 in addition to Supplementary Fig. 20.

### Durability of high-activity LaCoO_3_

**Fig. 7:**
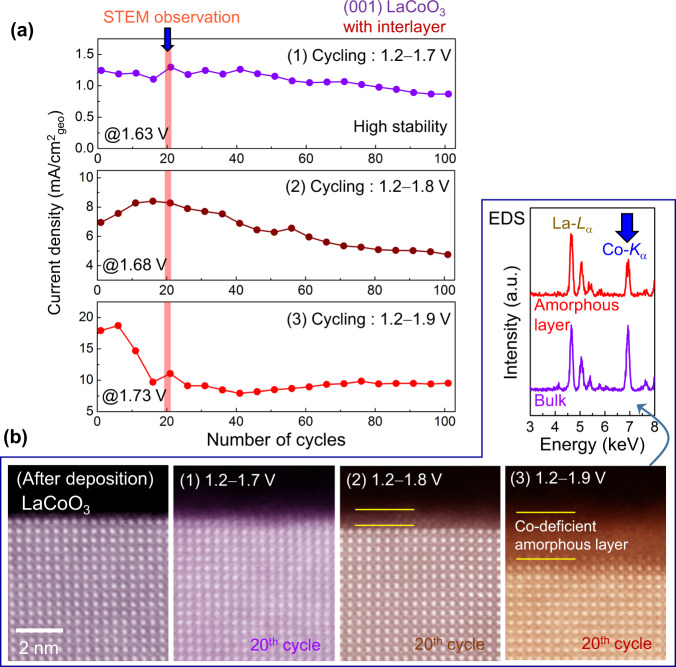
Durability of OER activity in LaCoO_3_ thin films. **a** A set of three plots for the current-density variation with anodic cycling is provided. If the potential range is adjusted so as not to exceed 1.7 V vs. RHE, the high OER current density of LaCoO_3_ is preserved even after 100 anodic cycles, demonstrating notable durability of the high activity. **b** A series of STEM images show the surface variations with different potential ranges during cycling. In addition to the image of a pristine film, each images was acquired after the 20th cycle, as indicated in red shadows in **a**. The formation of an amorphous layer is indicated by a pair of yellow lines in each image. As compared in the EDS spectra, this amorphization is induced by Co dissolution at high overpotential.

In addition to the distinct initial OER activities between LaCoO_3_ and LaNiO_3_, another property that should be mentioned is the durability of high catalytic activity. Based on previous reports on several oxides, it appears to be generally accepted that the stability of OER catalysts (or facets of a crystal) shows an inversely proportional correlation with the catalytic activity^34,52^. In this regard, we examined how long the high activity of LaCoO_3_ films could be preserved. Figure 7a presents the OER current densities of a pristine (001) LaCoO_3_ film with a conductive interlayer as a function of the anodic cycling number. It is noteworthy that three different potential ranges were utilized during cycling. As can be found in the first plot, its high activity (1 mA/cm^2^) does not substantially deteriorate even after 100 cycles in a range of 1.2–1.7 V vs. RHE, whereas a metallic (001) LaNiO_3_ film shows a gradual and substantial decrease of the OER activity with cycling (see Supplementary Fig. 21a for comparison). In contrast, when a much higher overpotential is applied up to 1.9 V (the third plot in Fig. 7a), significant degradation of the OER current density is observable within 20 cycles.

Atomic-column-resolved STEM images in Fig. 7b directly reveal that a Co-deficient amorphous layer is induced by anodic cycling on the LaCoO_3_ film surface (see the EDS spectra), demonstrating the remarkable dependence of the amorphization on the potential range. As already observed in a previous report^31^ on LaNiO_3_ films showing Ni dissolution (also see Supplementary Fig. 21b, c for the Ni-deficient composition of a surface amorphous layer), Co cations at high overpotential appear to dissolve into an alkaline electrolyte during cycling as well, resulting in the formation of a Co-deficient amorphous phase (see Supplementary Fig. 22) and subsequent reduction of the OER activity with cycling in the range of 1.2–1.9 V. Although larger overpotential is required (e.g., 1.8 V) in (001) LaNiO_3_ films in order to obtain a high OER current density (1 mA/cm^2^) (see Supplementary Fig. 21a), much lower overpotential (1.7 V) is sufficient to preserve the same OER activity in (001) LaCoO_3_ films. Breaking the conventional wisdom regarding oxide-based OER catalysts, this notable advantage of Co in terms of durability thus opens various possibilities for new design of catalyst materials achieving both high activity and long-time stability.

## Discussion

Three sets of experiments in the present study provide several significant implications regarding the oxide-based catalysts for the OER. First, the electronic conduction through the catalysts appears to be much more impactful than what has been usually considered in overall OER catalysis. The one order-of-magnitude variation of the OER current density in the LaCoO_3_ (001) surface with and without the conductive interlayer in Fig. 5f directly emphasizes the importance of electronic conduction, including the electronic conductivity of catalysts, in OER electrocatalysis. To precisely evaluate the real OER activity of a nonmetallic catalyst, such as LaCoO_3_ in this work, two resistive factors should be taken into account. One is the Ohmic loss (*iR*_Ohmic_) caused by the nonmetallic behavior of a catalyst and the other is the contact resistance (*iR*_cont_) induced between the catalyst and the electrode. Unless these two resistive components are efficiently eliminated, a large amount of applied potential during the OER would be seriously dissipated without directly contributing to the OER. We thus used nanoscale thin films to reduce *iR*_Ohmic_ and utilized a conductive interlayer deposited on the entire substrate to suppress *iR*_cont_. Under this sample configuration, we could successfully evaluate the notably high OER activity of LaCoO_3_ without interference of the two resistive components.

Second, Cr, the oxide form of which is known as one of the inactive transition-metal oxides for the OER, can be effective in addition to Fe, if the total composition of catalysts is optimized so that the high electrical conduction of charge is attained during the OER (note that the experimentally obtained bandgaps of pristine LaCrO_3_ and LaFeO_3_ are very large, ~3.4 eV and ~2.3 eV, respectively)^53,54^. Indeed, recent reports dealing with Cr doping and solid solutions in various materials to enhance the OER catalysis^55–58^ agree very well with our observations and DFT calculation for the Cr 3*d* DOS. Third, from the Al doping results in Figs. 1 and 4, catalytically inactive metal ions with no electrons in the *d* orbitals can make a viable contribution to the OER catalysis. As their substitution may induce the local distortion of neighboring [NiO_6_] octahedra, the Ni *d*-orbital levels become nondegenerate, resulting in the DOS variation^31,41^, as schematically illustrated in Supplementary Fig. 23a. Consequently, the density of neighboring Ni 3*d* states can change even by doping of catalytically inactive dopants. Indeed, we verified significantly different bond lengths in a neighboring [NiO_6_] octahedron of doped Al and subsequent distortion of the octahedron during the DFT calculation (see Supplementary Fig. 23b). In this respect, when a mixing approach with multiple components is utilized, a balanced consideration of the electronic structure on both the dopants and the matrix cation appears necessary for better understanding of catalysis enhancement.

Finally, the charge transfer from transition metals to adsorbates via oxygen (e.g., *M*^3+^−O(OH*)^−^ → *M*^4+^−OO*^2−^) during the OER on LaMO_3_ will be much easier and more likely to occur if a larger number of density of M 3*d* states are placed near the Fermi level rather than far below it. Consequently, Fe, Co, and Cr having substantial 3*d* states near the Fermi level appears to be considerably effective for the OER catalysis. However, please note that La*M*O_3_ (*M* = Fe, Cr, Mn, In, Sc) is a typical insulating oxide with a large bandgap (>2 eV), whereas LaCoO_3_ is semiconducting with a narrow bandgap (~0.5 eV). As a consequence, when Fe, Mn, In, and Sc were doped in metallic LaNiO_3_ films, we could observe a serious reduction of the electronic conductivity (see Supplementary Fig. 24). Therefore, in the case of Fe doping, it appears that there is a trade-off between the benefit of Fe doping and the disadvantage from the detrimental conductivity in terms of the concentration, demonstrating that 5% is the optimum concentration.

We have investigated the intrinsic contribution of the 3*d*-orbital states of various metal dopants to the OER activity on the La*M*O_3_-type perovskite surface. For a consistent and precise comparison, a crystallographically identical (001) surface was measured with a small amount of the dopants in metallic LaNiO_3_ and semiconducting LaCoO_3_ heteroepitaxial thin films with a conductive interlayer to exclude electronic conduction constraints. Consistent with the DOS at the (001) surface acquired by the DFT calculations, Co, Fe, Cr, and even Al were directly identified to be effective elements to enhance the OER catalysis in LaNiO_3_. In particular, the OER activity of Co was proved to be one order higher than Ni in the (001) La*M*O_3_-type perovskite surface together with notably better stability, in contrast to the conventional wisdom. Our study emphasizes that the 3*d*-orbital states near the Fermi level have a notable influence on the OER catalysis and also how seriously misleading the intrinsic OER activity of transition metals in oxides can be if the catalysts are not metallic.

## Methods

### Thin-film fabrication and X-ray analysis

Heteroepitaxial LaNiO_3_ and LaCoO_3_ thin films were prepared by using a sol–gel process. Starting materials for the preparation of precursor solutions were La(NO_3_)_3_ ∙ 6H_2_O (99.999%, Sigma Aldrich), Ni(CH_3_COO)_2_ ∙ 4H_2_O (99.998%, Sigma Aldrich), and Co(CH_3_COO)_2_ ∙ 4H_2_O (99.999%, Alfa Aesar). For doping experiments, Al(NO_3_)_3_ ∙ 9H_2_O (99.997%, Sigma Aldrich), Sc(NO_3_)_3_ ∙ 4H_2_O (99.9%, Sigma Aldrich), Mn(CH_3_COO)_2_ ∙ 4H_2_O (99.99%, Sigma Aldrich), Fe(NO_3_)_3_ ∙ 9H_2_O (99.95%, Sigma Aldrich), Cr(NO_3_)_3_ ∙ 9H_2_O (99.99%, Sigma Aldrich), and In(NO_3_)_3_ ∙ 3H_2_O (99.99%, Sigma Aldrich) were used as dopants. Each of the starting materials were first dissolved in 2-methoxyethanol (99.9%, Sigma Aldrich) and refluxed at 80 °C for 1 h under a constant stirring condition to prepare homogeneous precursor solutions with 0.2 M. Each of the precursor solutions was deposited on (001) SrTiO_3_ single-crystal substrates by a spin-coating method at 5000 r.p.m. for 10 s. The wet films were dried at 150 °C for 10 min on a hot plate, subsequently heat-treated at 400 °C for 10 min for pyrolysis, and finally annealed at 800 °C for 1 h in air for crystallization. A 5%-Fe-doped LaNiO_3_ epitaxial film was deposited on each SrTiO_3_ single-crystal substrate in an identical manner as a conductive metallic interlayer to facilitate the electron conduction to the film. The epitaxy of the grown films was confirmed by X-ray diffractometry (X’Pert-PRO MRD, PANalytical) with Cu-*K*_α_ radiation and direct STEM observation. The X-ray RSMs for the (103) reflections from the substrates and the films were acquired by using the same diffractometer.

### STEM, EDS, EELS, XPS, and AFM

Samples for all the STEM analyses were fabricated by focused ion-beam system (Helios Nanolab 450 F1, Thermo Fisher Scientific). Protective amorphous carbon and thin Pt layers were deposited on the epitaxial films before ion-beam milling. To minimize the sidewall damage and sufficiently thin the specimens for electron transparency, final milling was conducted at a voltage of ~2 kV. HADDF and BF-STEM images were taken with a transmission electron microscope (Titan cubed G2 60–300, Thermo Fisher Scientific) at 300 kV with a spherical aberration corrector (CEOS GmbH). The optimum size of the electron probe was ~1 Å with a convergence semiangle of 24 mrad. Chemical mapping with EDS was carried out in the Titan cubed G2 at 300 kV along with four integrated silicon-drift EDS detectors (ChemiSTEM™ technology) at a collection solid angle of 0.7 srad. La-*L*_α_ (4.6 keV), Ni-*K*_α_ (7.5 keV), Co-*K*_α_ (6.9 keV), Fe-*K*_α_ (6.4 keV), Al-*K*_α_ (1.5 keV), Mn-*K*_α_ (5.9 keV), Sc-*K*_α_ (4.1 keV), In-*L*_α_ (3.3 keV), Sr-*L*_α_ (1.8 keV), and Ti-*K*_α_ (4.5 keV) lines were selected during elemental mapping. The probe current was adjusted to be 50−100 pA with a scanning time of <300 s. The EDS maps were low-pass filtered using Bruker ESPRIT software after the reduction of background noise for better visualization. As the Cr-*K*_α1_ (5.42 keV) and La-*L*_β2_ (5.38 keV) lines seriously overlap with each other, EELS analysis was performed for Cr mapping with a Gatan Image Filter (GIF Quantum 965, Gatan, Inc.). Electron energy-loss spectra for the Cr-*L* edges were acquired for spectrum imaging with a dispersion of 0.25 eV per channel and a collection aperture of 5 mm in diameter. The valence state of dopants was investigated using an X-ray photoelectron spectroscope (K-Alpha XPS, Thermo Scientific) with monochromatic Al-*K*_α_ radiation (1486.7 eV) and flood gun emission of 150 μA. The Ni 2*p*, Co 2*p*, Cr 2*p*, Al 2*p*, Sc 2*p*, Mn 2*p*, and In 3*d* peaks were compared with those from the reference crystals of a DyScO_3_ single crystal, Cr_2_O_3_ polycrystals, In_2_O_3_ polycrystals, a LaAlO_3_ single crystal, and LaMnO_3_ polycrystals. No significant chemical shift or broadening of the peaks was found, verifying the trivalent state of the dopants. In addition, because the photoemission Fe 2*p*_1/2_ and 2*p*_3/2_ peaks seriously overlap with the Ni Auger peaks in XPS, an EELS analysis was performed by using LaFeO_3_ polycrystals as a reference to examine the valence state of Fe. The surface topology of each film was examined by an atomic force microscope (Cypher VRS, Oxford Instruments) with a Si probe (tip radius of curvature: ~7 nm, AC160TS-R3, Oxford Instruments) in non-contact tapping mode.

### DFT calculations

Ab initio DFT calculations for DOS of oxygen 2*p* and metal *p* and *d* orbitals at the (001) surface of doped LaNiO_3_ were carried out using the spin-polarized local density approximation (LDA) functional for exchange correlation, along with the ultrasoft pseudopotentials for ionic cores, as implemented in the CASTEP code (Biovia Inc.). A sufficiently long (001)-surface slab along with a 10-Å vacuum layer was constructed as an optimum supercell for each calculation to make the relaxation layer of each slab more than 10 Å in thickness. To account for the electron localization around the transition-metal ions, the LDA + *U* method with the Hubbard *U* parameter (4.0 eV for Ni 3*d*, Co 3*d*, and Fe 3*d* states; 2.0 eV for Mn 3*d* states; 3.5 eV for Cr 3*d* states) was employed^59–62^. Low-spin (*t*_2g_^6^)(*e*_g_^1^) for *d*^7^ Ni^3+^, intermediate-spin (*t*_2g_^5^)(*e*_g_^1^) for *d*^6^ Co^3+^, high-spin (*t*_2g_^3^)(*e*_g_^2^) for *d*^5^ Fe^3+^, high-spin (*t*_2g_^3^)(*e*_g_^1^) for *d*^4^ Mn^3+^, and high-spin (*t*_2g_^3^)(*e*_g_^0^) for *d*^3^ Cr^3+^ configurations were assumed, respectively^61^. The plane-wave basis set for the kinetic energy cutoff was 500 eV. Relaxation of the internal coordinates for each atom was performed using the Broyden–Fletcher–Goldfarb–Shanno algorithm with convergence tolerances of 0.1 eV/Å for the maximum ionic force, 5 × 10^−5^ eV/atom for the total energy, and 0.005 Å for the maximum ionic displacement.

### Electrochemical and electrical tests and measurements

All electrochemical reactions and measurements were conducted with a potentiostat (Biologic SP-300) in a 0.1 M KOH aqueous solution (pH 12.9) prepared by using the Milli-Q water (18.2 MΩ·cm) and KOH pellets (Sigma Aldrich, 99.99%) to achieve sufficiently high purity. A Pt counter electrode and a saturated Ag/AgCl reference electrode were used. The measured potential values vs. the Ag/AgCl reference electrode were converted into the RHE scale by using the following equation at 25 °C,

1 $$E_{{\mathrm{RHE}}} = E_{{\mathrm{Ag}}/{\mathrm{AgCl}}} + 0.059 \cdot {\mathrm{pH}} + E^\circ _{{\mathrm{Ag}}/{\mathrm{AgCl}}}$$

where *E*_RHE_ is the converted potential vs. RHE, *E*_Ag/AgCl_ is the measured potential against the Ag/AgCl reference electrode, and *E*°_Ag/AgCl_ is the standard potential of Ag/AgCl (KCl, 3 M) at 25 °C, i.e., 0.21 V. For thin-film samples, the substrate and the connecting copper wire were completely covered with chemically inert insulating epoxy resin (Henkel Loctite^®^ EA 9460) after application of silver paint (Cans Elcoat P-100) on the back side of a thin-film sample so as to expose the film surface only. All electrolyte solutions were presaturated by bubbling O_2_ for 30 min under constant O_2_ bubbling. Cyclic potential was applied to the samples at a rate of 10 mV/s, sweeping from 1.2 to 1.75 V vs. RHE to record the first-cycle OER current density in each case. During the durability tests of thin films, the current densities were measured in various cyclic potential ranges up to 1.9 V. Samples were taken out of the electrolyte solution in order to remove O_2_ gas bubbles adsorbed on the film surface every five anodic cycles during the durability tests. Electrochemical impedance spectroscopy was also carried out in the same potentiostat in a frequency range from 0.1 Hz to 1 MHz with an amplitude of 10 mV for *iR* correction of the uncompensated series resistance. DL capacitance was measured to examine the variation of the electrochemical active surface area between thin-film samples. The DL capacitance measurements were carried out in a non-faradaic potential range of 1.03–1.17 V vs. RHE by recording the current values as a function of scan rate. To precisely present the OER current values on the basis of the DL capacitance, the converted plots showing the OER current normalized by the DL capacitance are provided in Supplementary Fig. 25. A two-point probe method along with Pt electrodes was utilized to measure the sheet resistance of thin-film samples. The thickness of each thin-film sample was directly measured by STEM observation.

## Supplementary information

Supplementary Information

## Data Availability

The data that support the findings of this study are available from the corresponding author (S.-Y.C.) upon reasonable request.
