# Supplementary material for: Elucidating intrinsic contribution of d-orbital states to oxygen evolution electrocatalysis in oxides
Source: Nat Commun. 2021 Feb 5;12:824. doi: 10.1038/s41467-021-21055-0 (PMC7865077; doi:10.1038/s41467-021-21055-0)
Supplement: Supplementary file 1 — Supplementary Information [file 41467_2021_21055_MOESM1_ESM.pdf]

Supplementary Information

Elucidating intrinsic contribution of *d*-orbital states to oxygen evolution electrocatalysis in oxides

Yun et al.

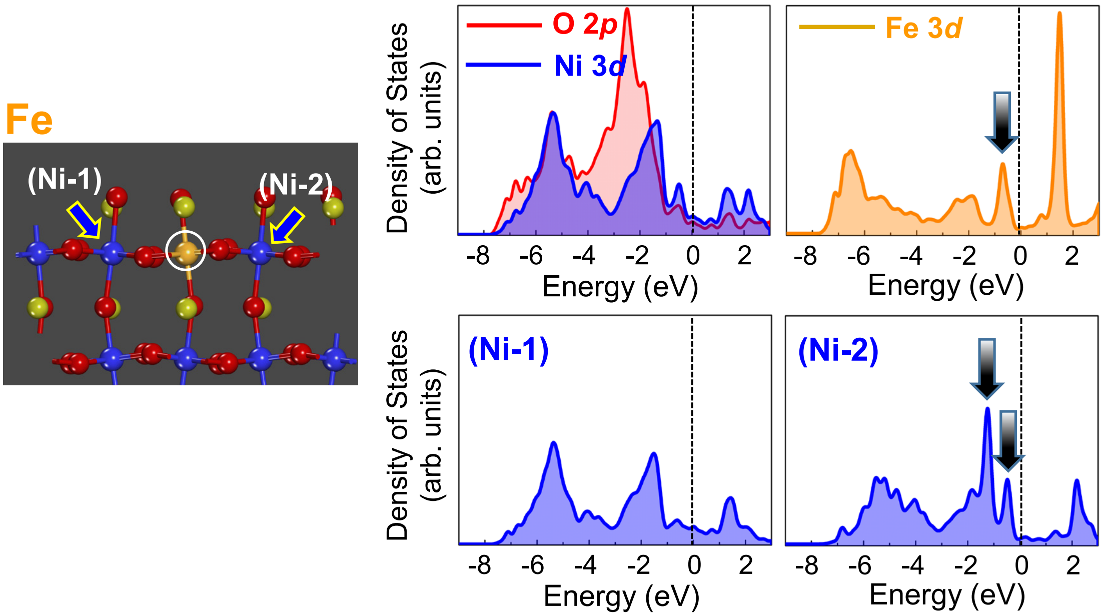

Supplementary Fig. 1 DOS at the $LaNiO_3$ $(001)_{cubic}$ surface with Fe. As indicated by a pair of black arrows in the plot for Ni-2, Fe doping induces significant increment of the neighboring Ni 3*d* DOS near the Fermi level in addition to the high density of Fe 3*d* states between −2 and 0 eV.

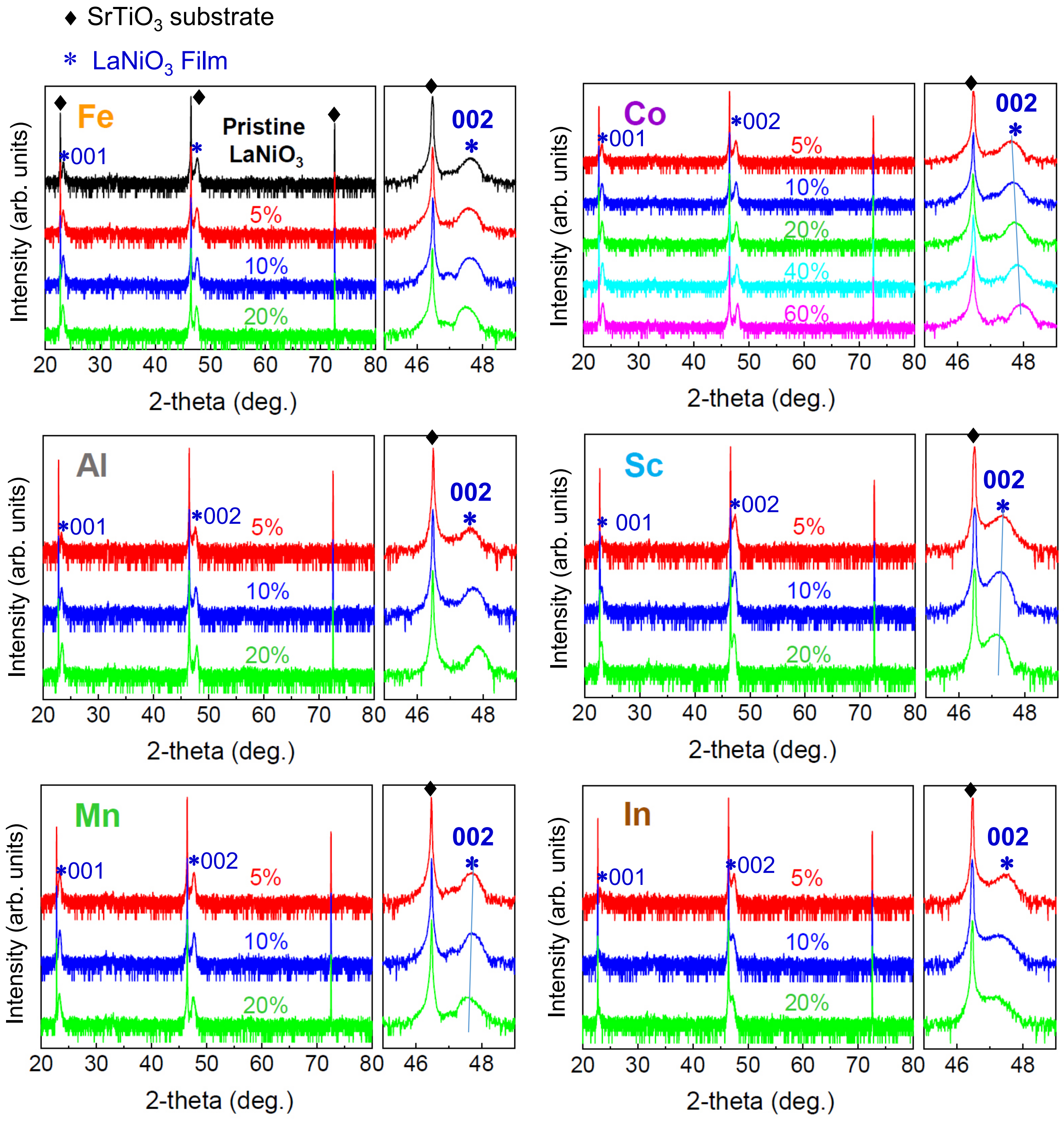

Supplementary Fig. 2 X-ray diffraction patterns of $LaNiO_3$ thin films with dopants. As denoted by asterisks, the appearance of the (00*l*) Bragg reflections from each film verifies the heteroepitaxial growth of the films on (001) $SrTiO_3$ single-crystal substrates. In addition, a consistent peak shift with increasing doping concentration up to 20% (even 60% in the case of Co doping) indicates that each dopant is completely soluble into the $LaNiO_3$ lattice.

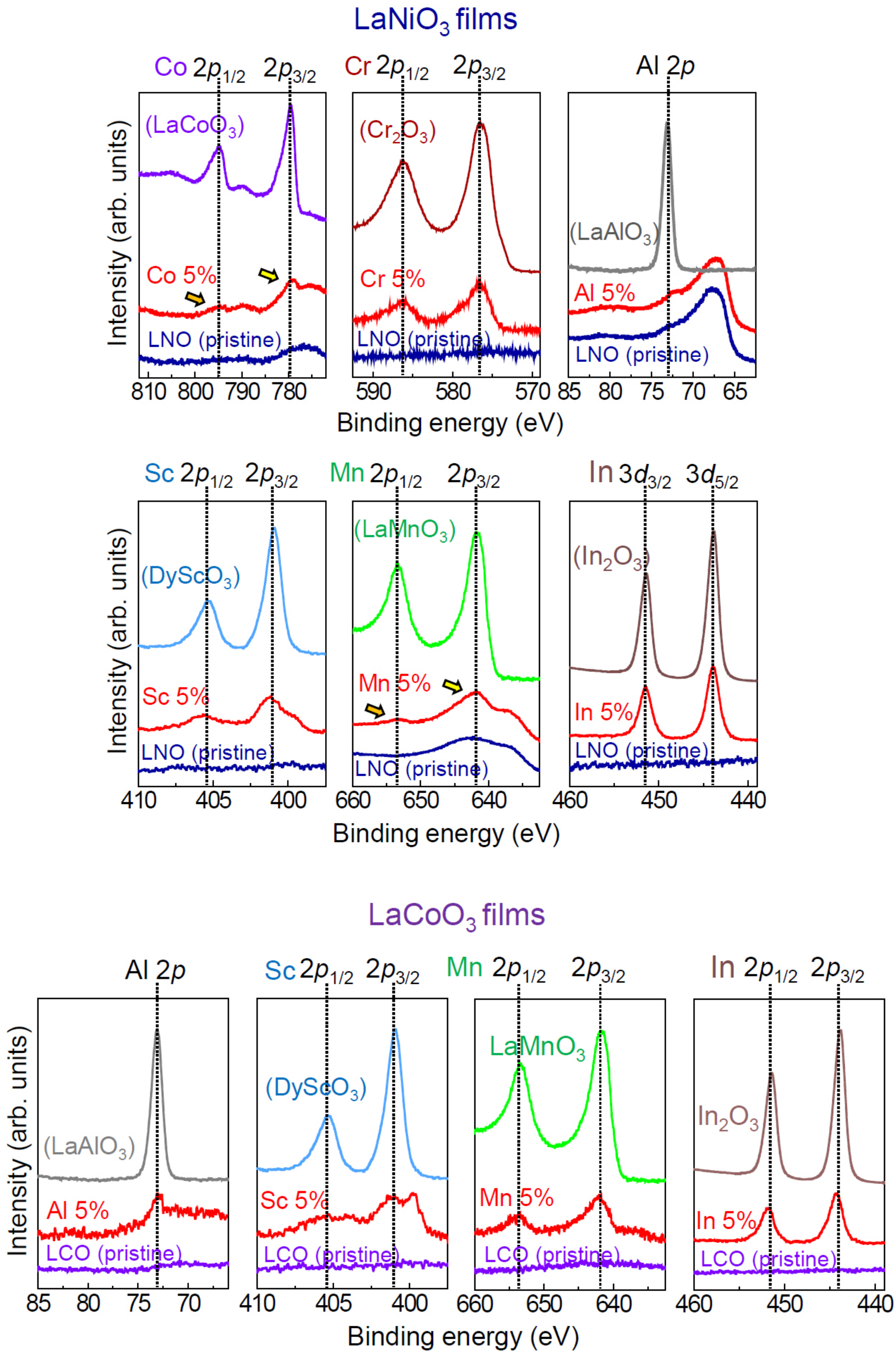

Supplementary Fig. 3 XPS analysis. To verify the valence state of each dopant, an XPS analysis was carried out by using a $DyScO_3$ single crystal, Cr_2O_3 polycrystals, In_2O_3 polycrystals, a $LaAlO_3$ single crystal, and $LaMnO_3$ polycrystals as reference crystals for comparison. As shown in this series of spectra, the valence state of each dopant was confirmed to be the same as that of each reference crystal (3+).

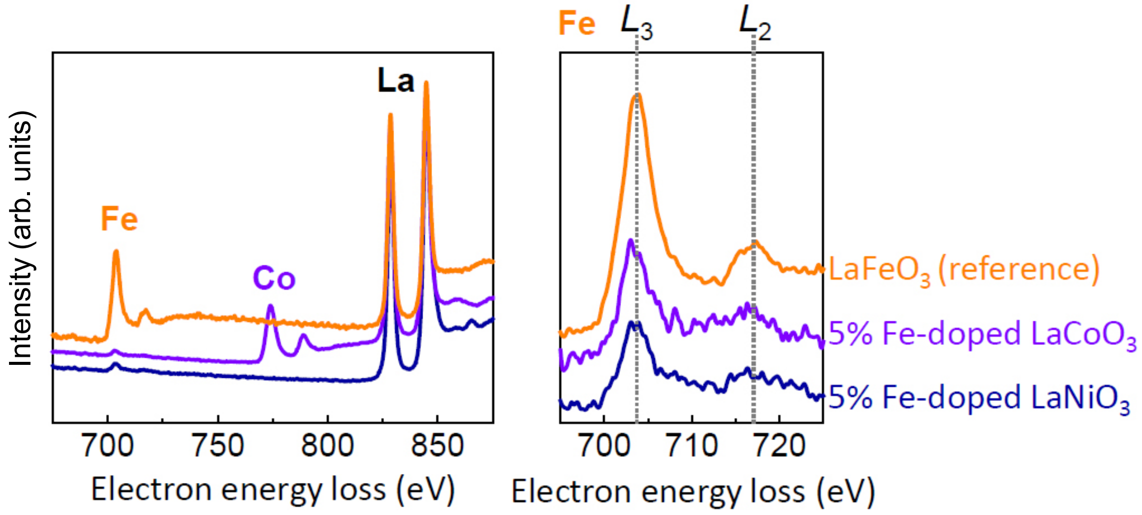

Supplementary Fig. 4 EELS results. Because the photoemission Fe $2p_{1/2}$ and $2p_{3/2}$ peaks seriously overlap with the Ni Auger peaks in XPS, an EELS analysis was performed instead by using $LaFeO_3$ polycrystals as a reference to examine the valence state of Fe doped in $LaNiO_3$ and $LaCoO_3$ films. The peak positions of Fe L-lines from the thin-film samples agree well with those of the trivalent Fe from the reference crystals.

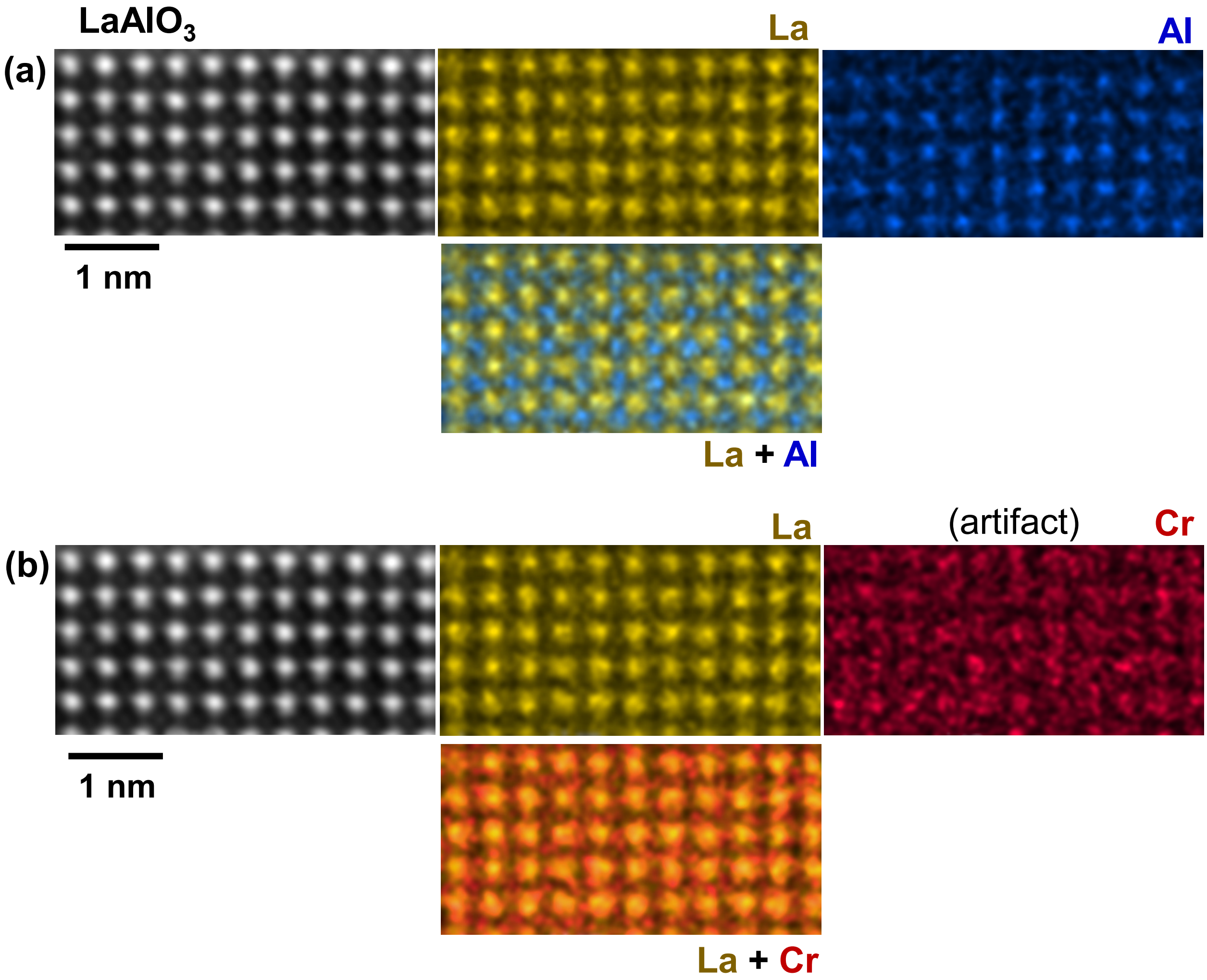

Supplementary Fig. 5 EDS analysis for Cr. The position of the Cr-$K_{\alpha 1}$ peak (5.42 keV) is very close to that of the La-$L_{\beta 2}$ peak (5.38 keV) in the EDS spectrum. Consequently, the utilization of EDS is not appropriate for Cr detection when La-containing compounds are analyzed. To clarify this problematic issue, two series of atomic-column-resolved EDS maps obtained from a $LaAlO_3$ single crystal with no Cr are exemplified. (**a**) This set of EDS maps for La, Al, and La+Al clearly discriminates the La site and the Al site in the $LaAlO_3$ lattice, as the Al-K_α (1.5 keV) peak does not overlap with any other La peaks in the EDS spectrum. (**b**) In contrast, when an X-ray signal at 5.42 keV (the position of Cr-$K_{\alpha 1}$) is recorded during the EDS of $LaAlO_3$, a sufficiently detectable intensity from the shoulder of the La-$L_{\beta 2}$ peak (5.38 keV) can be obtained. This artifact may be misinterpreted as a Cr signal despite the absence of Cr impurity in the single-crystal $LaAlO_3$ specimen.

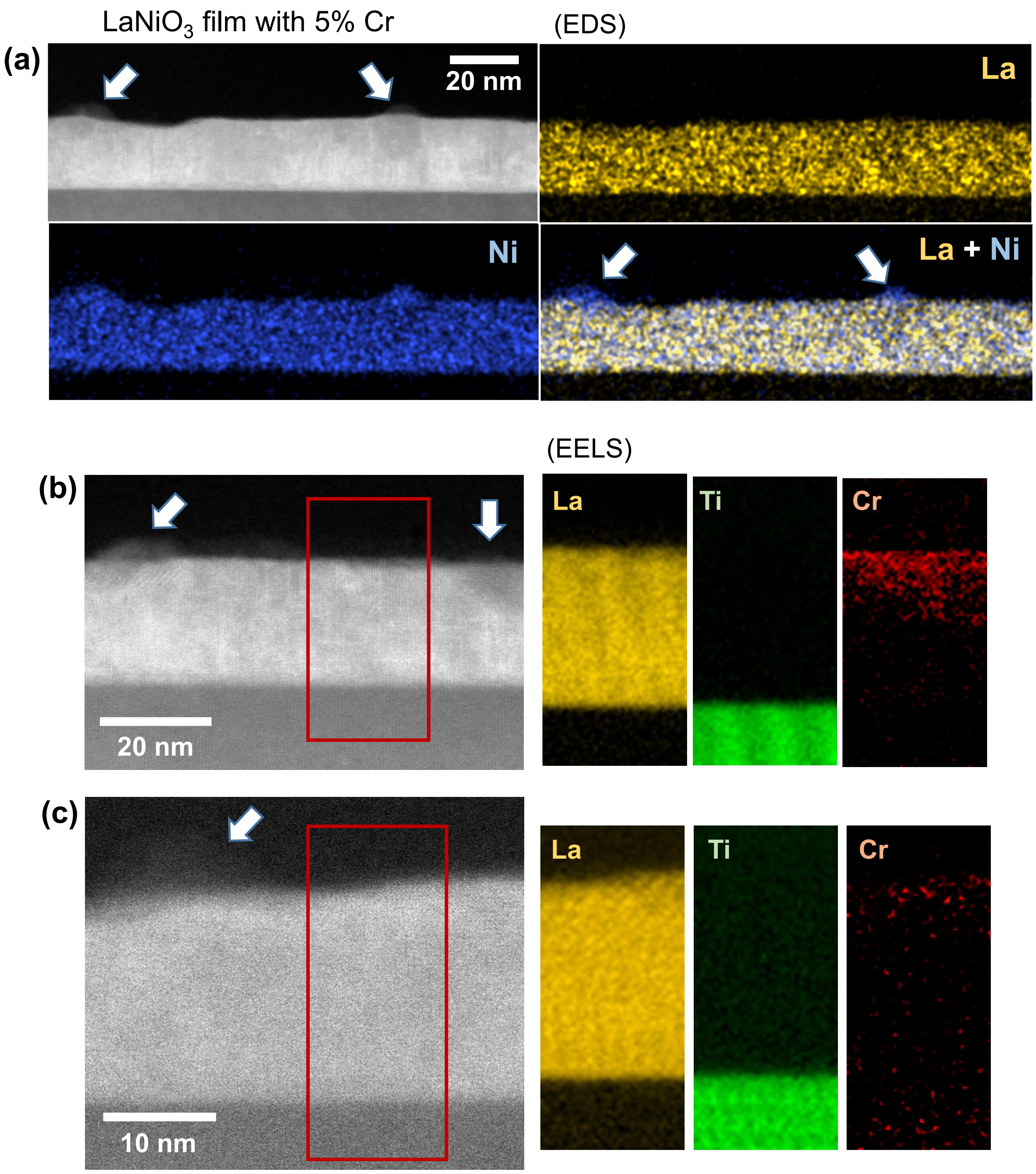

Supplementary Fig. 6 EDS and EELS analyses for Cr-doped $LaNiO_3$ thin films. (**a**) As indicated by white arrows, Ni-rich secondary impurity phases are frequently observed at the surface of Cr-doped $LaNiO_3$ thin films during the EDS analysis. (**b**,**c**) Two sets of EELS maps demonstrate the seriously inhomogeneous distribution of Cr in the film. While strong Cr segregation at the film surface is identified in the first set (b), no significant Cr signal is detected in the second set (c). White arrows indicate the Ni-rich impurity phases.

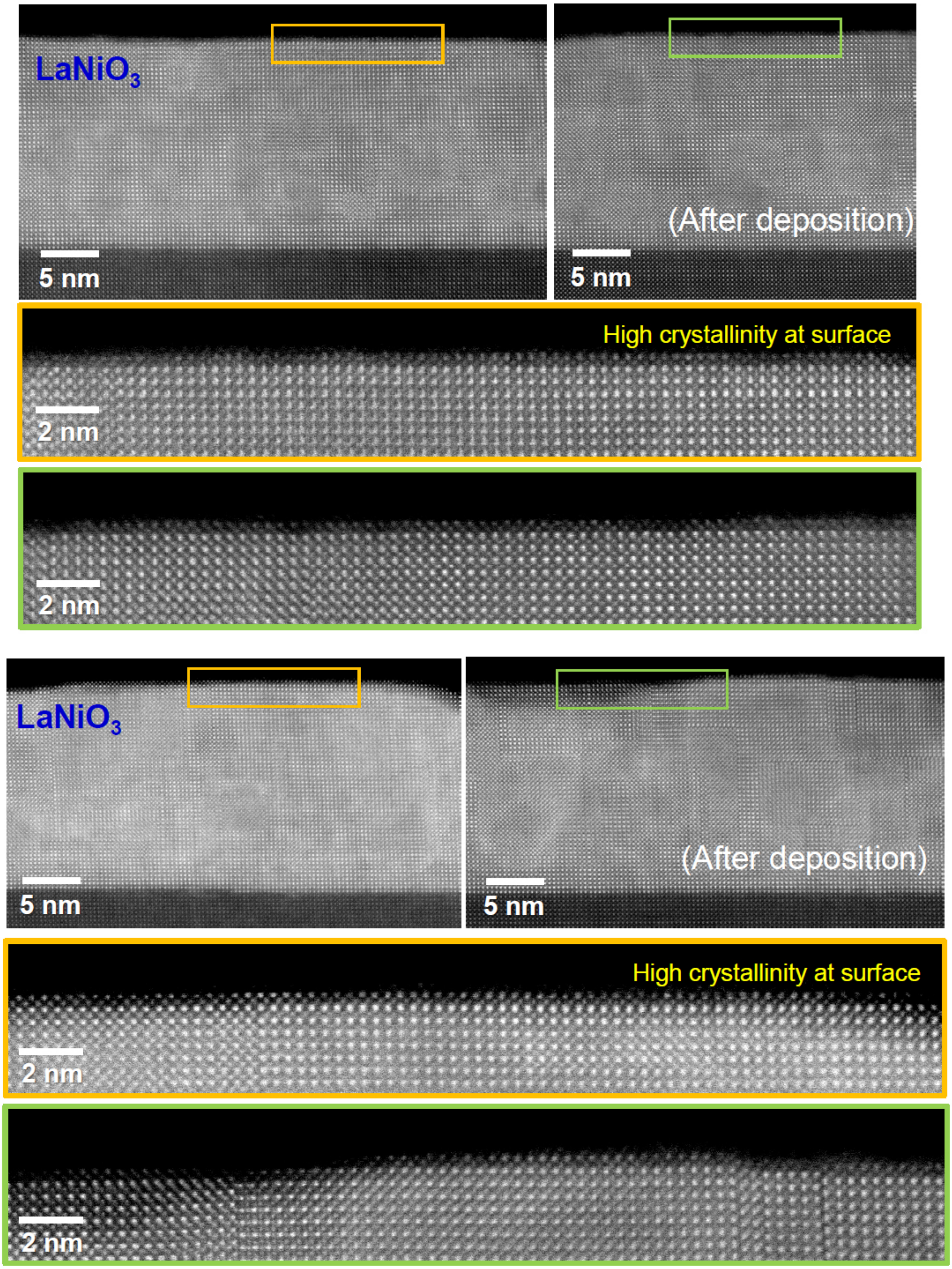

Supplementary Fig. 7 HAADF STEM images of pristine $LaNiO_3$ thin films after deposition. As clarified in each enlargement, high crystallinity with neither secondary impurities nor amorphous phases on the surface is verified.

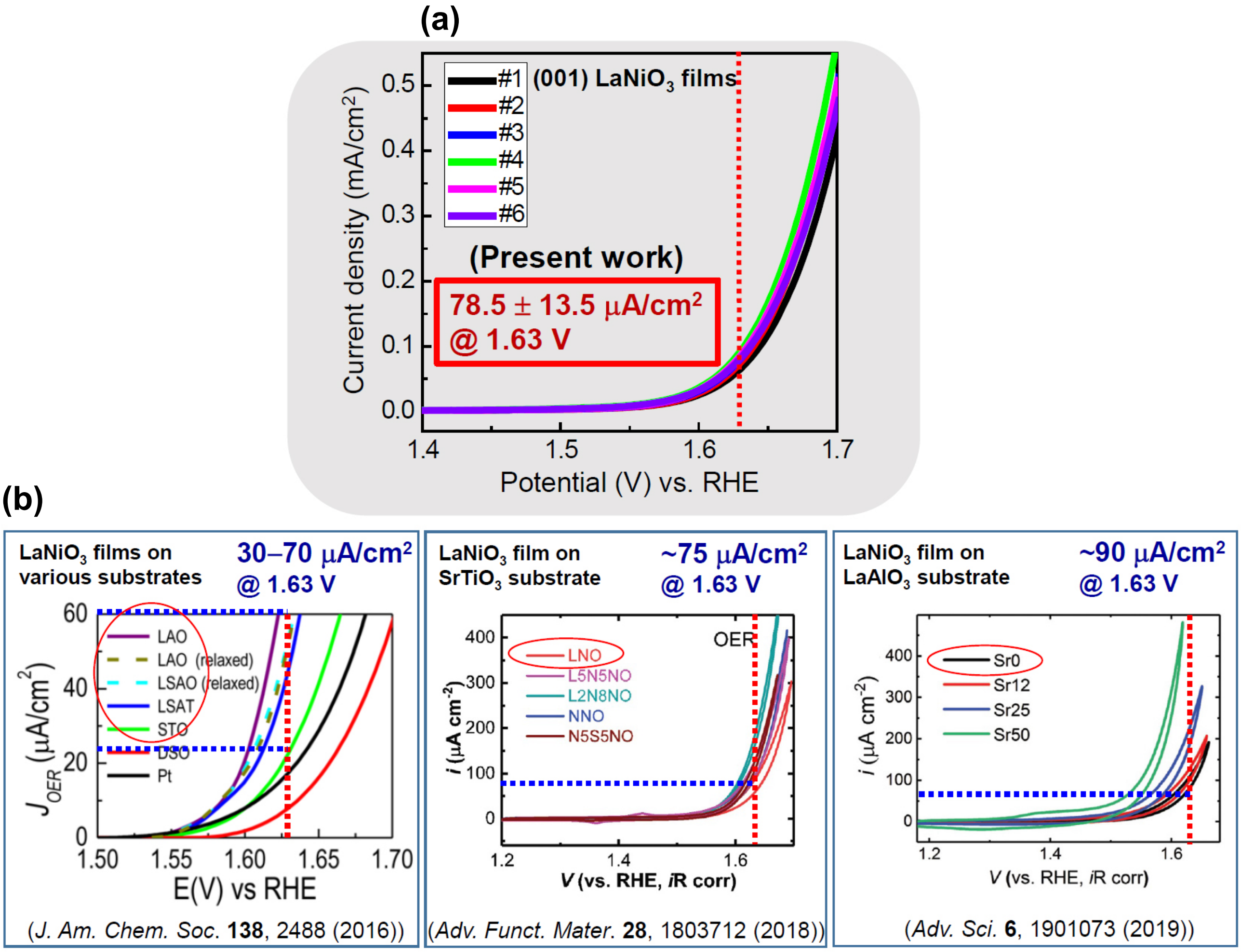

Supplementary Fig. 8 Comparison of OER current densities in $LaNiO_3$ thin films. (**a**) The OER activity of our six pristine (001) $LaNiO_3$ films fabricated in an identical manner in this work is 78.5 μA/cm^2 on average. (**b**) These activity values are comparable with the current densities (30–90 μA/cm^2) of (001) $LaNiO_3$ films reported in previous studies. The graphs are reproduced from refs. 42–44 with permission. Copyright 2016 American Chemical Society (ref. 42); copyright 2018 Wiley-VCH (ref. 43); copyright 2019 Wiley-VCH (ref. 44).

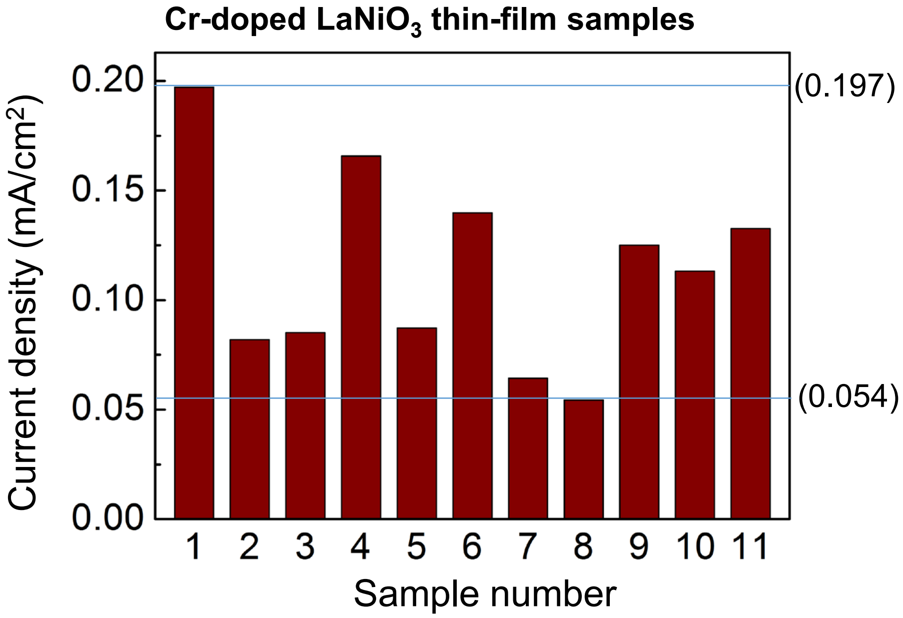

Supplementary Fig. 9 OER current densities of Cr-doped $LaNiO_3$ thin films. Eleven Cr-doped $LaNiO_3$ thin-film samples were prepared in an identical manner to investigate the variation of OER activity. As shown in this bar graph, the OER current density is noted to significantly vary among the samples, ranging from 0.054 to 0.197 mA/cm^2, although they were fabricated using the same method. The seriously inhomogeneous distribution of Cr is thus very likely to be responsible for the large variation of the OER activity. Nevertheless, the current density of most samples exceeds the value of the pristine $LaNiO_3$ sample, 0.075 mA/cm^2, demonstrating that Cr doping is beneficial to the OER catalysis.

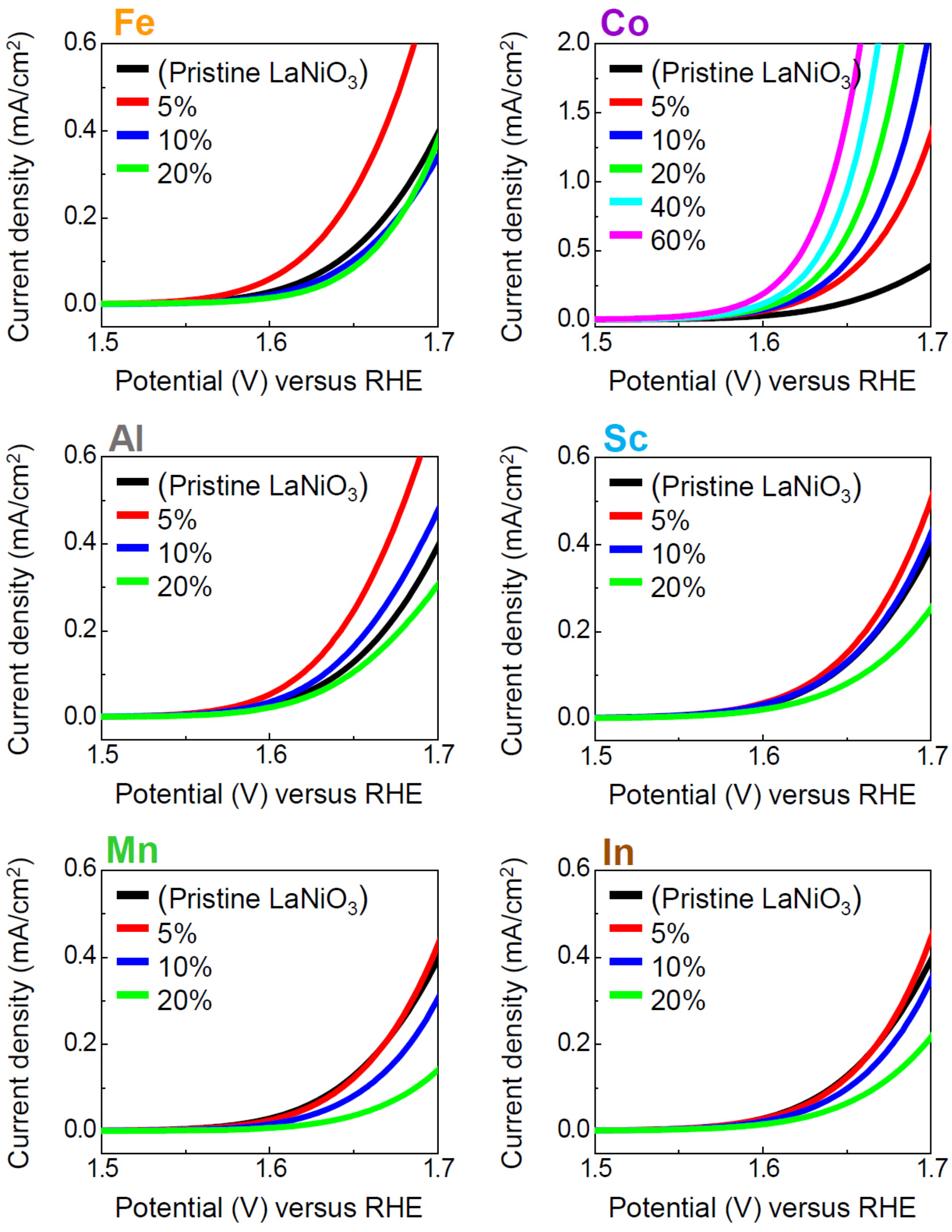

Supplementary Fig. 10 Plots for the OER current variations with doping concentration in each doped (001) $LaNiO_3$ sample. In contrast to other doped samples, Co shows a proportional correlation between the OER activity and the doping level up to 60%, while enhanced activity is observed at 5% doping in many other cases. The current-density values measured at 1.63 V vs. RHE are presented for comparison in the bar graph in Figure 4(b) in the main text.

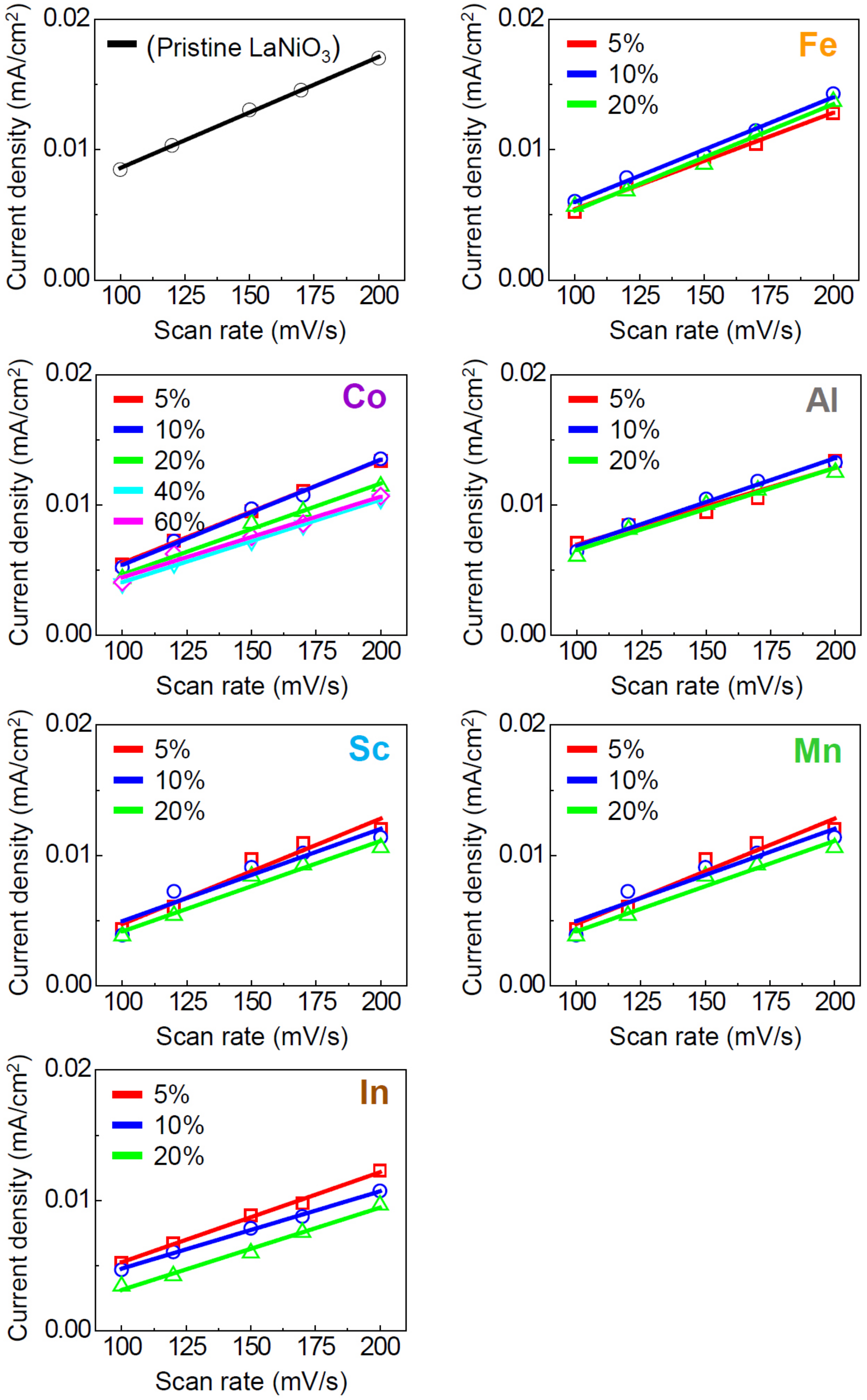

Supplementary Fig. 11 DL capacitance measurements of $LaNiO_3$ thin films with dopants. Cyclic voltammograms were acquired in a non-faradaic potential region (1.03−1.17 V vs. RHE) at different scan rates. The DL anodic charging currents are plotted as a function of scan rate in each case of doping. As can be observed in this series of plots, the DL capacitance values do not significantly vary among the thin-film samples, indicating a similar electrochemically active surface area. Specific values are summarized in Supplementary Table S1.

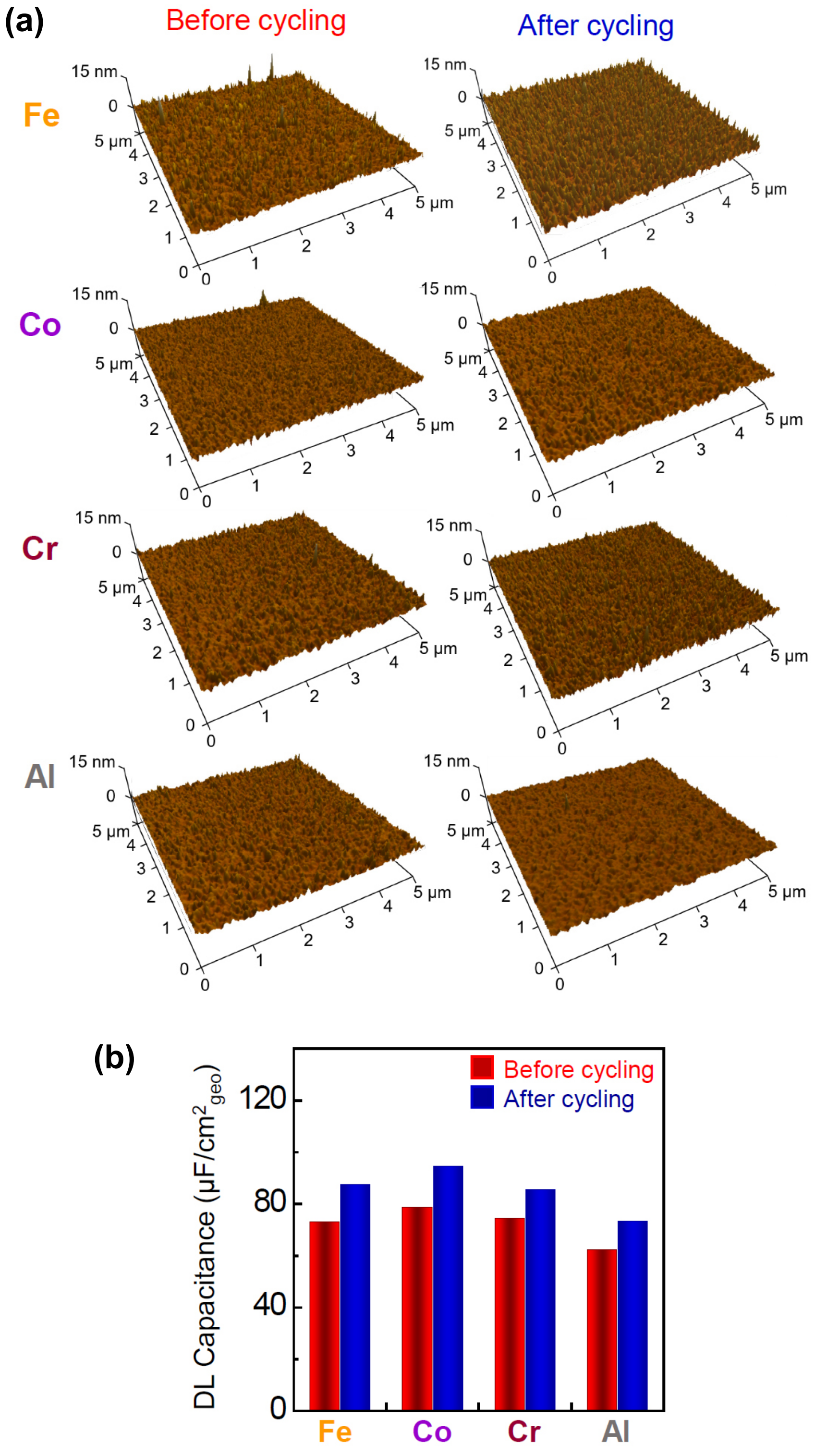

Supplementary Fig. 12 Surface topologies by AFM and DL capacitance variations of doped $LaNiO_3$ films before and after anodic cycling. (**a**) The surface topographic images confirm that the surface morphology and the roughness average of the samples do not significantly vary after OER cycling, in agreement with the DL capacitance variation. (**b**) Although the DL capacitance slightly increases after two cycles in each sample, the variation is merely ~15%. As a result, the notable enhancement of the OER activities by Fe, Co, Cr, and Al shown in Figure 4 in the main text stems from the doping effect rather than surface morphology variation.

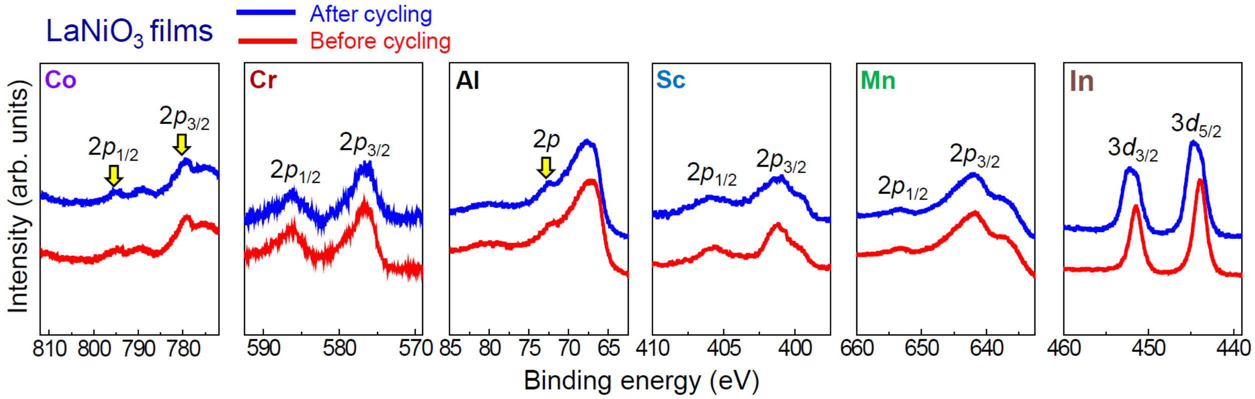

Supplementary Fig. 13 XPS of dopants in $LaNiO_3$ films. After OER cycling, nearly the same peak intensities of the dopants are verified, showing no serious dopant dissolution during the anodic cycles.

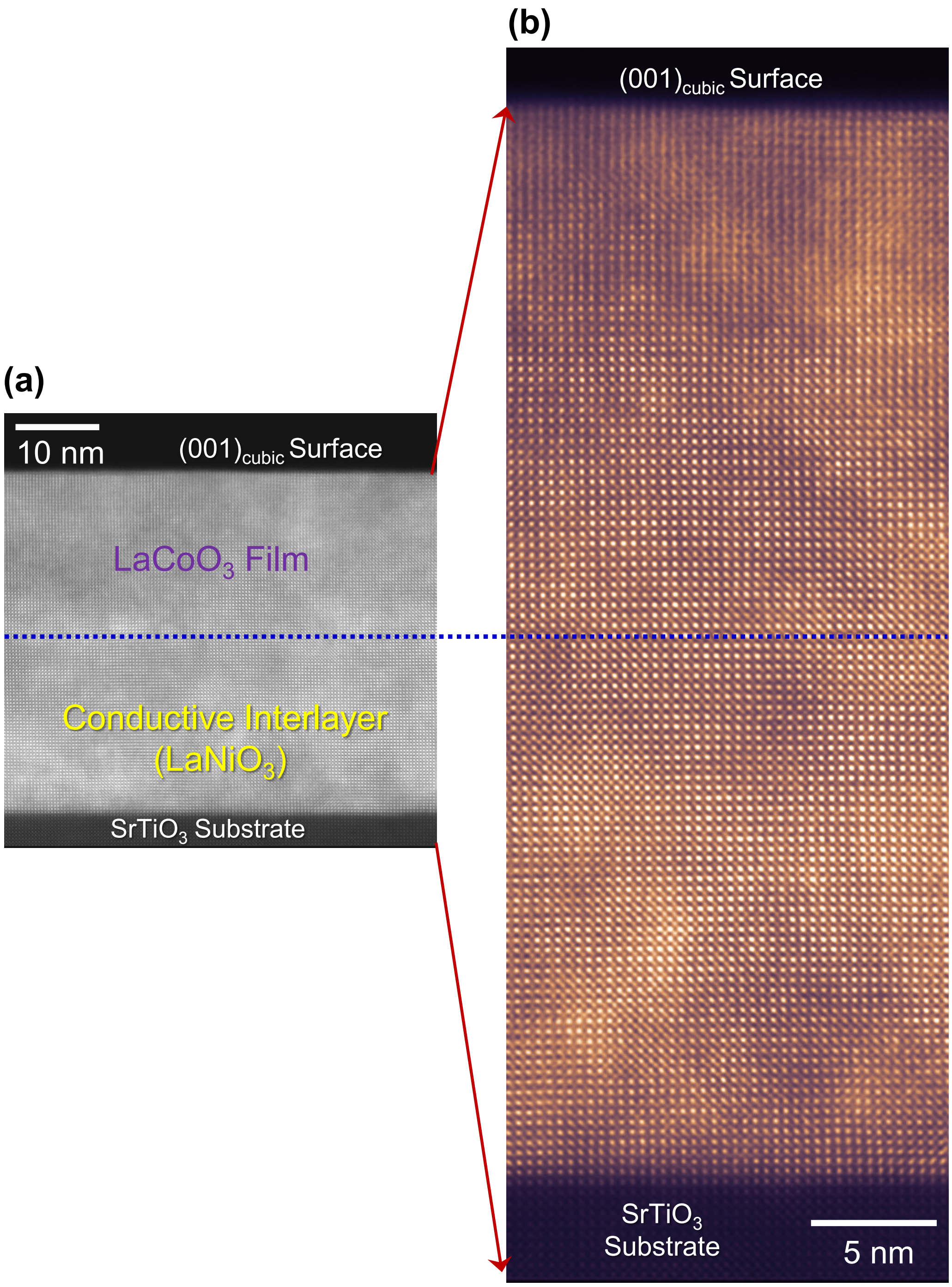

Supplementary Fig. 14 HAADF STEM images of a doped $LaCoO_3$ film with a conductive interlayer. (**a**) A low-magnification HAADF image was acquired from a Fe-doped $LaCoO_3$ film with a metallic $LaNiO_3$ interlayer on a $SrTiO_3$ substrate. (**b**) An entire cross-sectional image at a higher magnification (in color) is provided on the right-hand side. The heteroepitaxy between the $LaCoO_3$ film, the interlayer, and the substrate is directly verified by this atomic-column-resolved imaging.

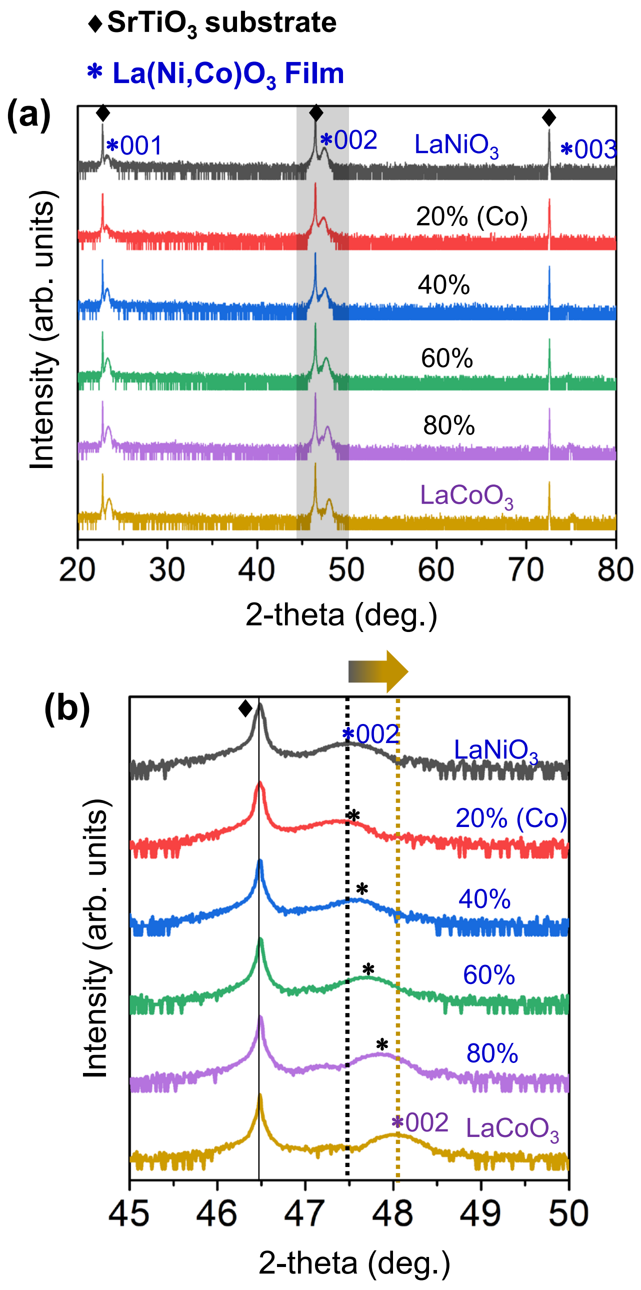

Supplementary Fig. 15 X-ray diffraction patterns of $La(Ni_{1-x}Co_x)O_3$ (x = 0–1) solid-solution thin films. All the thin films were directly deposited on (001) $SrTiO_3$ single-crystal substrates without an interlayer. (**a**) As denoted by blue asterisks, the appearance of the (00*l*) Bragg reflections from each film demonstrates the heteroepitaxial growth of the films on the substrates. (**b**) A series of diffraction patterns in the range of 2θ = 45°–50° is shown to clarify the consistent right-hand shift of the (002) peaks of the films (asterisks) with increasing Co content, while the position of the $SrTiO_3$ (002) peak (diamond) does not change. Therefore, a full range of solid solutions between $LaNiO_3$ and $LaCoO_3$ is verified.

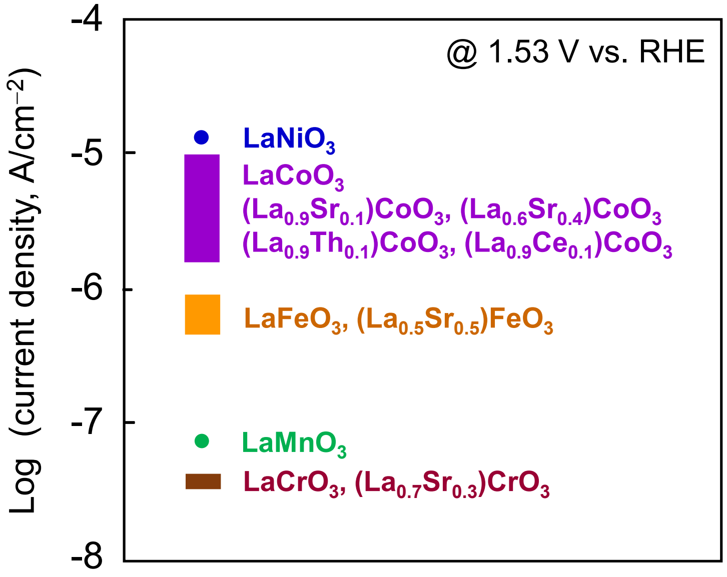

Supplementary Fig. 16 Comparison of the OER current densities of La-based perovskite catalysts. All the data are from the previous reports (refs. 11 and 12) by Bockris and Otagawa. Note that $LaNiO_3$ is reported to show the highest OER activity among many perovskite oxides in the previous studies.

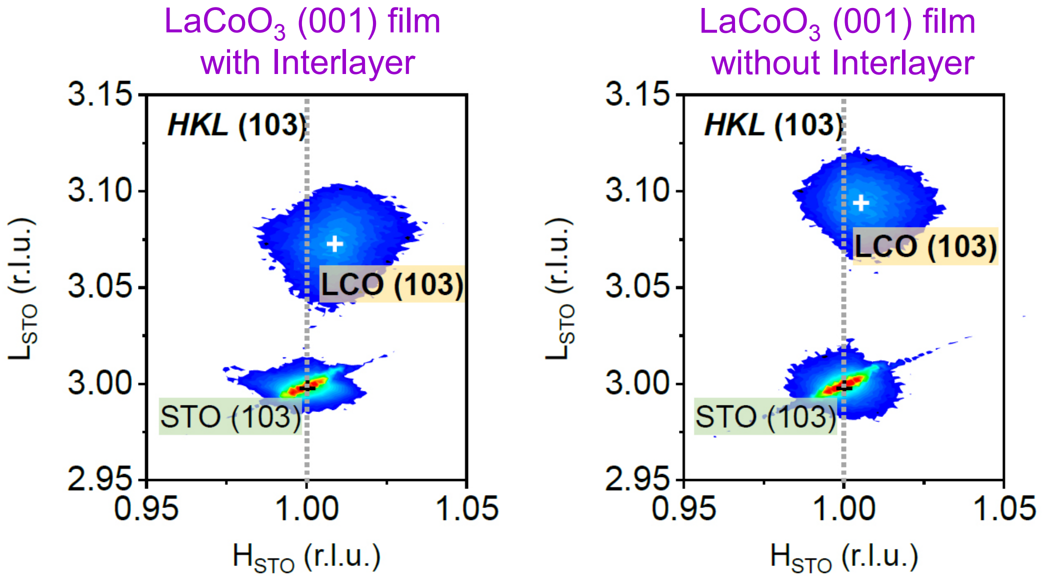

Supplementary Fig. 17 X-ray reciprocal space maps (RSM). In the cases of $LaCoO_3$ films grown on a $LaNiO_3$ conductive interlayer, the total thickness of the $LaCoO_3$ film and a $LaNiO_3$ interlayer is >50 nm, indicating that most strain is relaxed. This set of RMS clarifies the strain relaxation in the film on the $LaNiO_3$ interlayer. As a consequence, the remarkably high OER activity observed in the $LaCoO_3$ film with the $LaNiO_3$ interlayer is not related to the epitaxial strain.

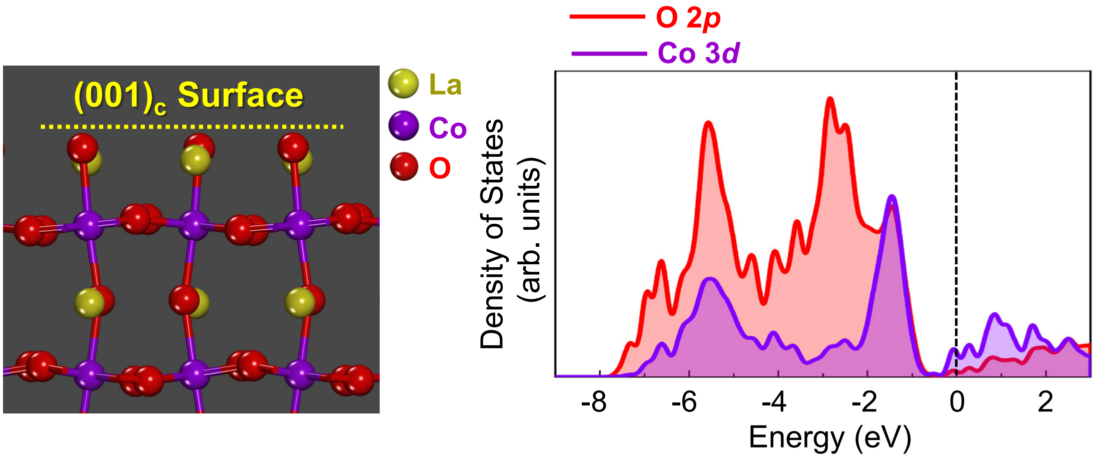

Supplementary Fig. 18 DOS at the pristine $LaCoO_3$ $(001)_{cubic}$ surface. The densities of the Co and O states were derived from the first octahedra at the surface. A high density of the 3*d* Co states is noted between −2 and 0 eV below the Fermi level.

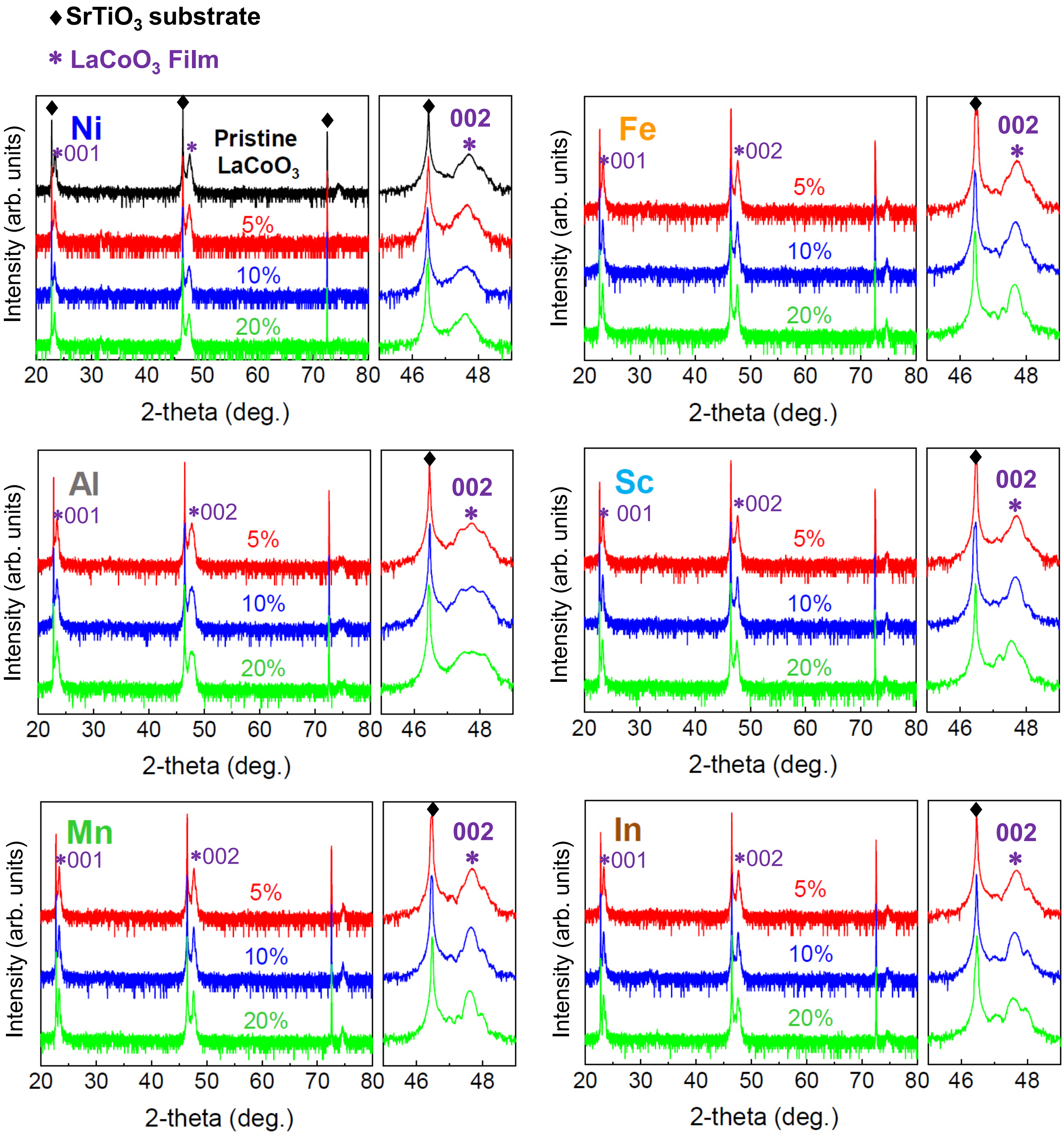

Supplementary Fig. 19 X-ray diffraction patterns of $LaCoO_3$ thin films with dopants. All the thin films were directly deposited on (001) $SrTiO_3$ single-crystal substrates without an interlayer for phase verification. As denoted by asterisks, the appearance of the (00*l*) Bragg reflections from each film demonstrates the heteroepitaxial growth of the films on substrates. A consistent peak shift with increasing doping concentration up to 20% also indicates that each dopant is completely soluble into the $LaCoO_3$ lattice.

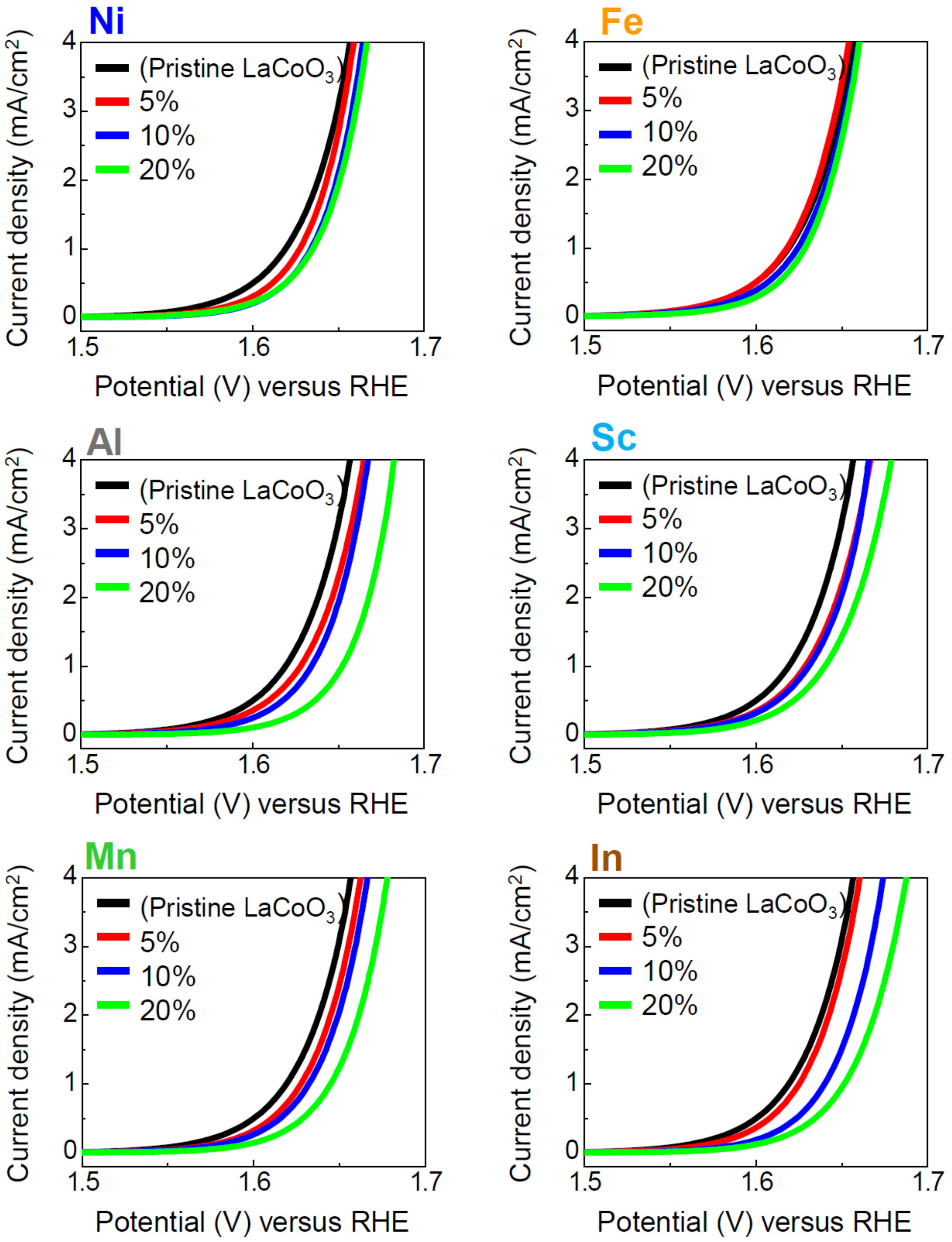

Supplementary Fig. 20 Plots for the OER current variations with doping concentration in each doped (001) $LaCoO_3$ sample. Decreasing OER activity with doping is observed in most samples, indicating that doping has a detrimental effect in $LaCoO_3$. The current-density values measured at 1.63 V vs. RHE are presented for comparison in the bar graph in Figure 6c in the main text.

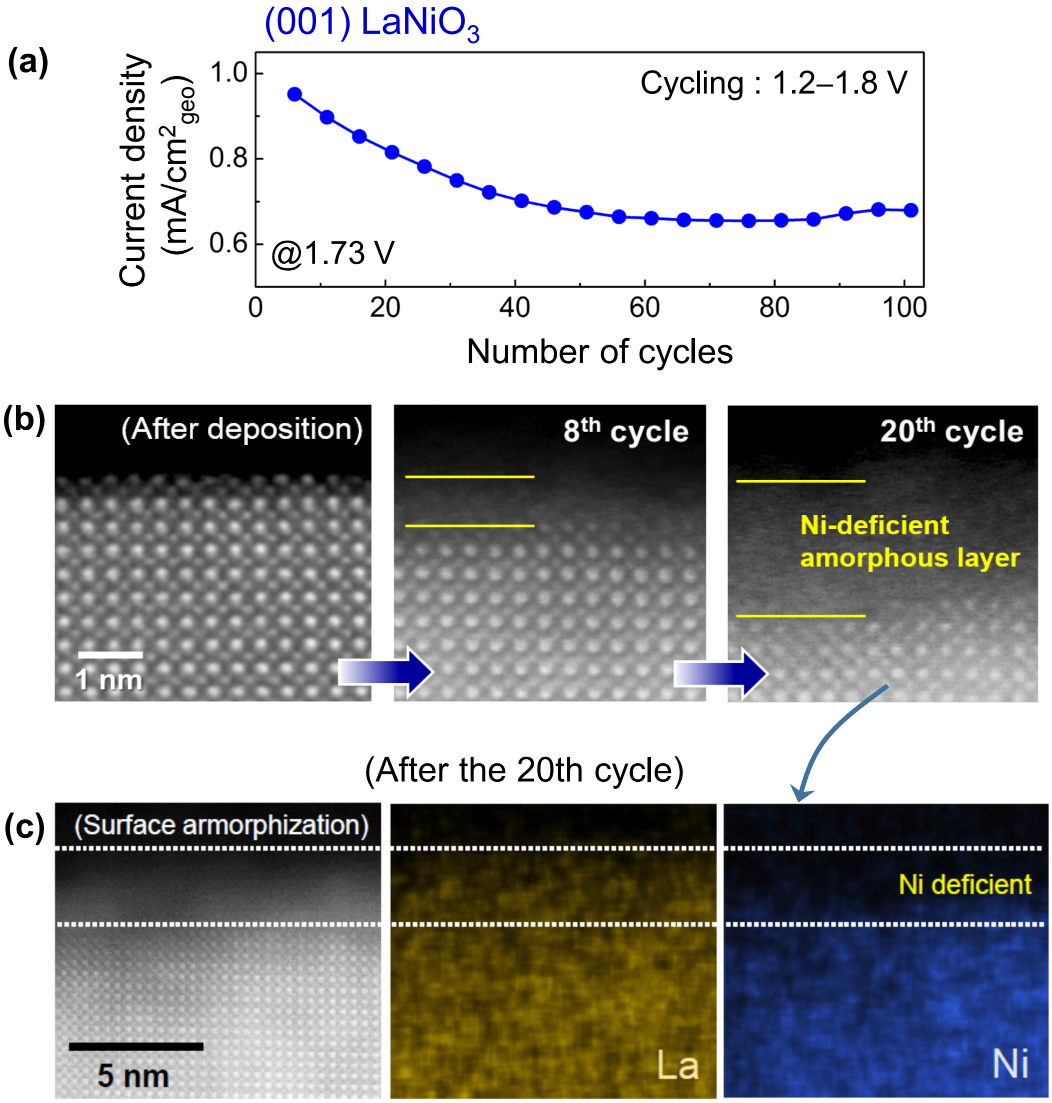

Supplementary Fig. 21 Durability of OER activity in $LaNiO_3$ thin films. (**a**) Gradual degradation of the OER current density is observed, when anodic cycling is carried out in a range of 1.2–1.8 V vs. RHE. (**b**) The film surface becomes amorphous with cycling, demonstrating that this amorphization is induced by high anodic potential. (**c**) A set of EDS maps acquired after 20 anodic cycles clearly shows that the surface amorphous layer on the film is Ni- deficient. Consequently, Ni dissolution into the electrolyte during cycling appears to be responsible for the surface amorphization.

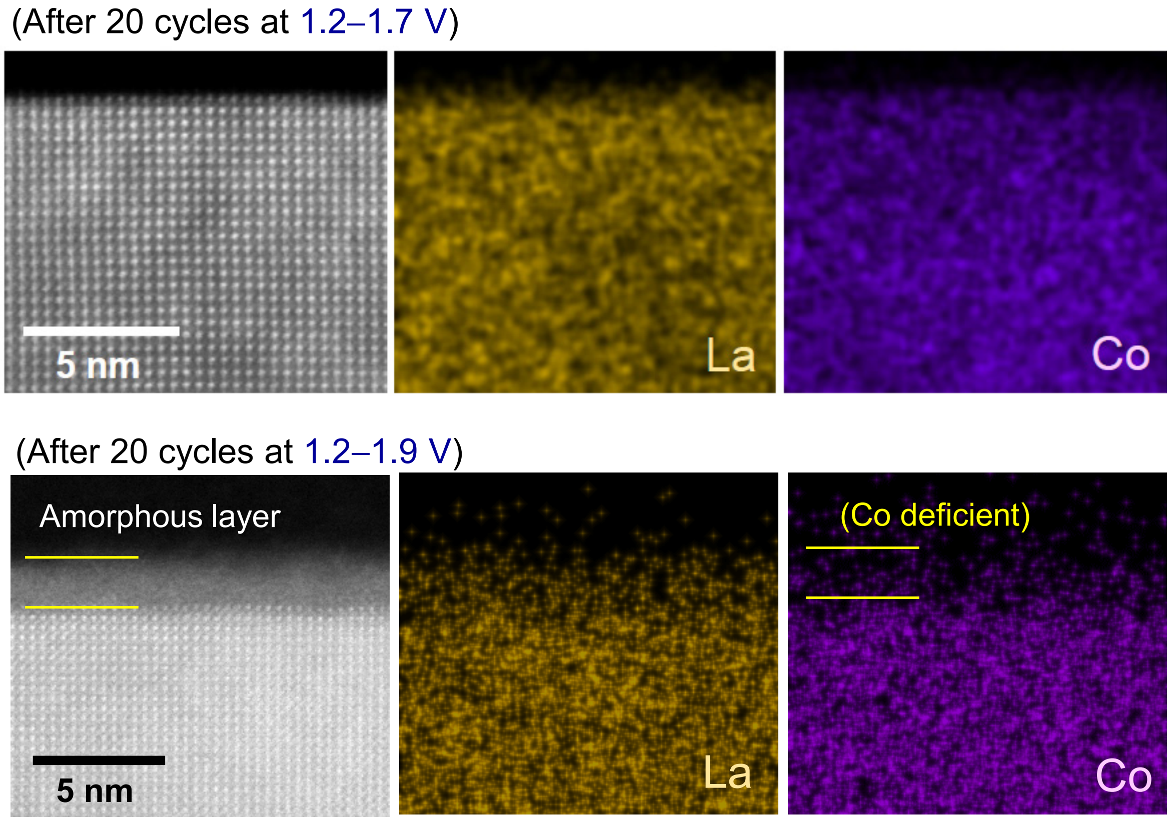

Supplementary Fig. 22 Comparison of the surface structure. Anodic-cycling-induced amorphization is observed in $LaCoO_3$ as well, when a large overpotential is applied during cycling. The formation of a surface amorphous layer is identified and the extent of amorphization depends on the potential range for the OER. No substantial amorphous layers are observable after 20 cycles, when anodic cycling is carried out in a range of 1.2–1.7 V vs. RHE. In contrast, if a much higher overpotential is applied up to 1.9 V, the generation of a surface amorphous layer is clearly observed, showing a Co deficiency in the composition. This amorphization in $LaCoO_3$ is thus very likely to be induced by Co dissolution, as consistently identified in $LaNiO_3$ by Ni dissolution.

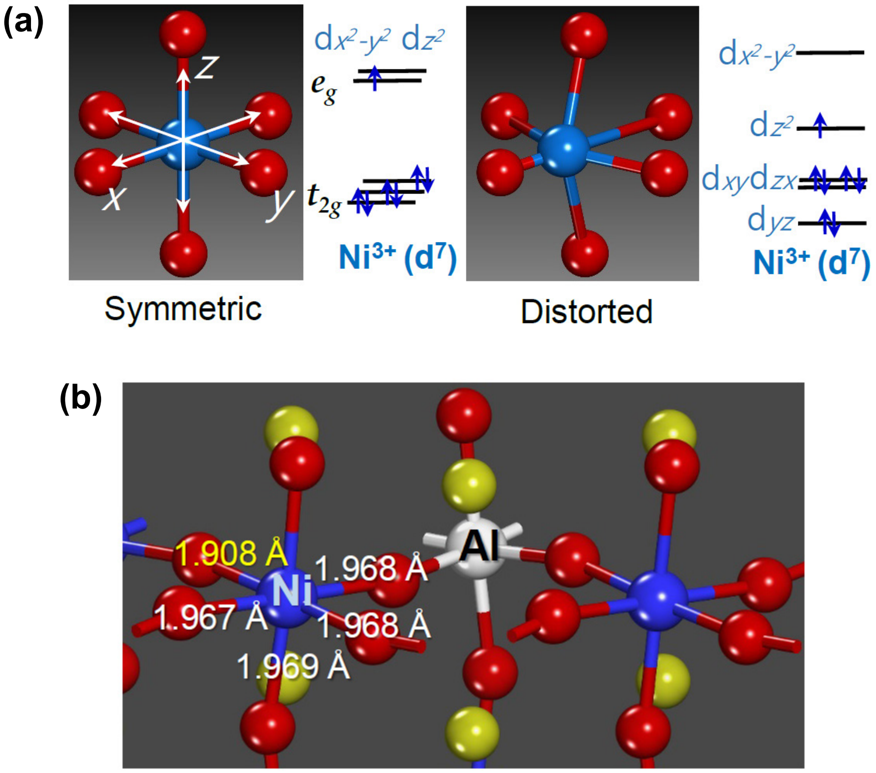

Supplementary Fig. 23 Ni 3*d*-orbital level nondegeneracy induced by symmetry breaking. Although Al is catalytically inactive, its substitution can induce local distortion of neighboring [NiO_6] octahedra, resulting in nondegenerate *d*-orbital levels, as schematically illustrated in (**a**). Consequently, the density of neighboring Ni 3*d* states can change even by doping of catalytically inactive Al, substantially contributing to increment of the DOS near the Fermi level. (**b**) Significantly different bond lengths are verified in a neighboring [NiO_6] octahedron of doped Al and subsequent distortion of the octahedron during the DFT calculation.

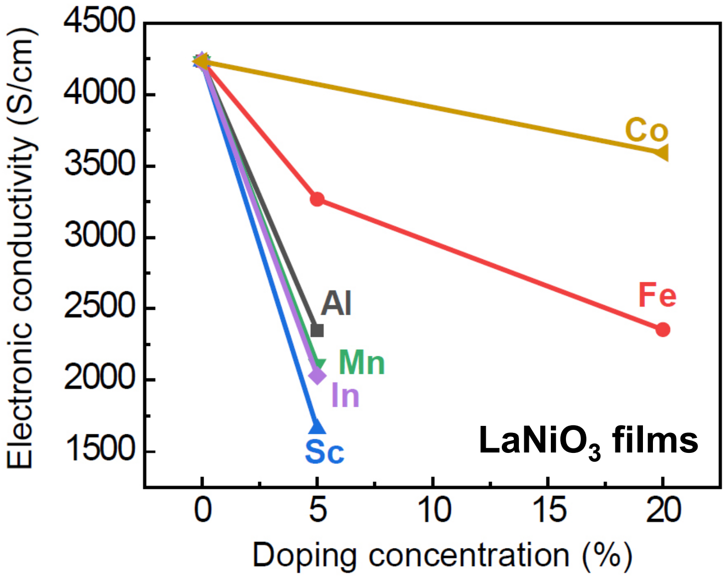

Supplementary Fig. 24 Electronic conductivity variation with doping in $LaNiO_3$ films. It is noted that LaMO_3 (M = Fe, Cr, Al, Mn, In, Sc) is a typical insulating oxide with a large bandgap (>3 eV), while $LaCoO_3$ is semiconducting with a narrow bandgap (~0.5 eV). As a consequence, when Fe, Mn, Al, In, and Sc are doped in metallic $LaNiO_3$ films, serious reduction of the electronic conductivity is observed.

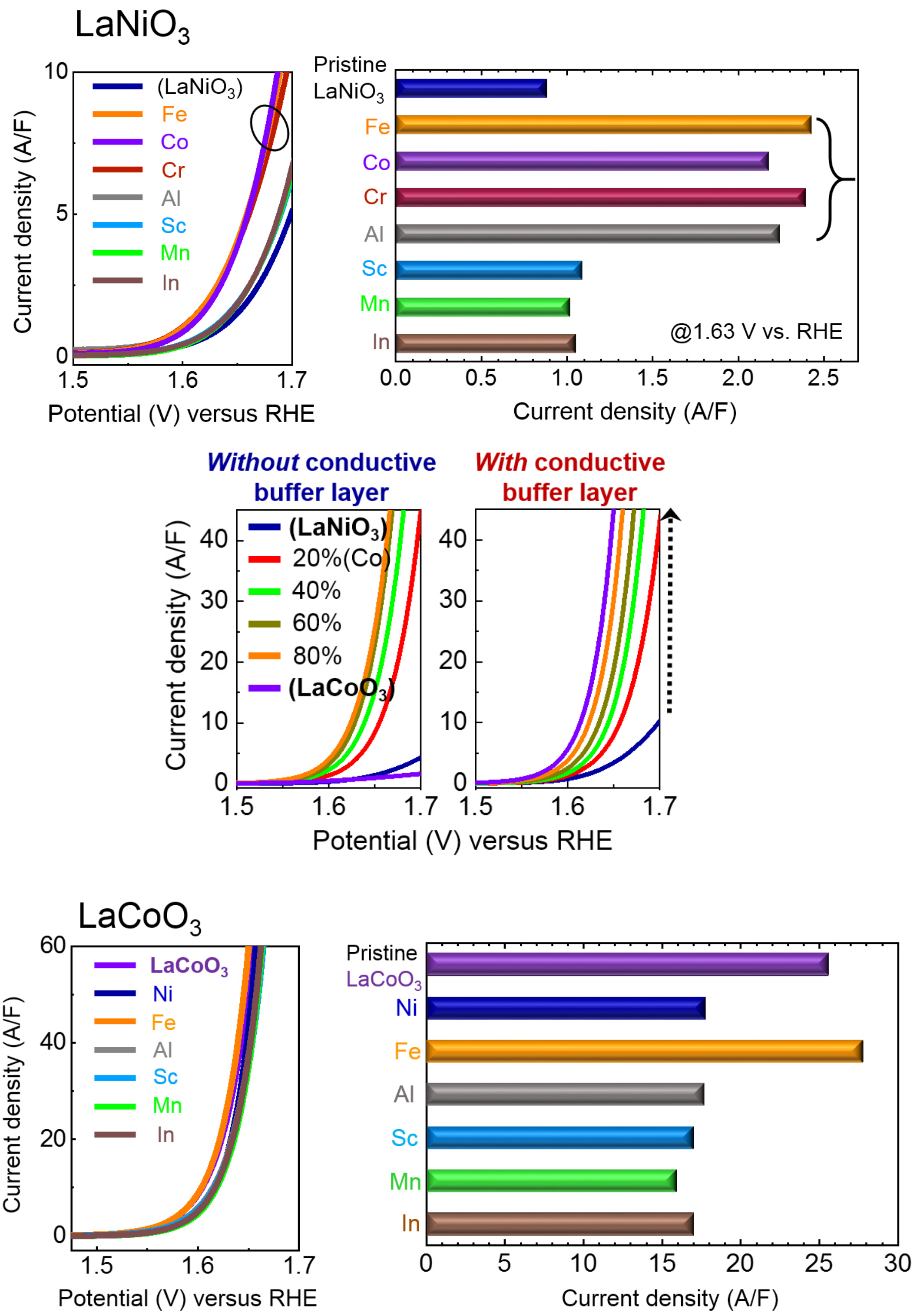

Supplementary Fig. 25 Plots of the OER current values normalized by the DL capacitance. It is noted that remarkable enhancement of OER activity by doping of Fe, Co, Cr, and Al in $LaNiO_3$ is consistently presented in this series of plots.

Supplementary Table 1. Current densities, Tafel slopes, and DL capacitances of doped $LaNiO_3$ thin films

		Current density at 1.63 V vs. RHE [mA/cm^2]	Tafel slope [mV/dec]	DL Capacitance [µF/cm^2]
$LaNiO_3$		0.075	78.4	90.3
(Dopant) Al	5%	0.141	71.7	62.3
	10%	0.094	74.8	67.7
	20%	0.061	75.5	58.3
Sc	5%	0.087	81.4	80.3
	10%	0.077	84.7	67.8
	20%	0.049	87.6	68.5
Mn	5%	0.068	73.4	66.7
	10%	0.048	73.1	61.4
	20%	0.020	76.1	47.0
Fe	5%	0.150	71.4	73.1
	10%	0.058	77.0	81.1
	20%	0.044	70.1	80.6
Co	5%	0.177	70.7	78.9
	10%	0.213	65.8	77.8
	20%	0.287	59.6	69.7
	40%	0.411	56.1	63.9
	60%	0.660	56.7	62.4
In	5%	0.070	79.2	66.8
	10%	0.056	79.9	59.3
	20%	0.038	84.5	58.3

Supplementary Table 2. Current densities, Tafel slopes, and DL capacitances of $La(Ni_{1-x}Co_x)O_3$ (x = 0–1) solid-solution thin films without conductive interlayer

**Without Interlayer**	**Current density at 1.63 V vs. RHE [mA/cm^2]**	**Tafel slope [mV/dec]**	**DL Capacitance [µF/cm^2]**
**$LaNiO_3$**	0.075	78.4	91.3
**Co 20%**	0.261	63.2	63.9
**Co 40%**	0.435	60.4	60.1
**Co 60%**	0.561	58.1	52.0
**Co 80%**	0.808	61.0	64.5
**$LaCoO_3$**	0.050	108	67.6

Supplementary Table 3. Current densities, Tafel slopes, and DL capacitances of $La(Ni_{1-x}Co_x)O_3$ (x = 0–1) solid-solution thin films with conductive interlayer

**With Interlayer**	**Current density at 1.63 V vs. RHE [mA/cm^2]**	**Tafel slope [mV/dec]**	**DL Capacitance [µF/cm^2]**
**$LaNiO_3$**	0.145	71.6	73.5
**Co 20%**	0.315	62.2	80.8
**Co 40%**	0.416	59.2	73.1
**Co 60%**	0.600	58.0	71.1
**Co 80%**	0.822	56.3	61.7
**$LaCoO_3$**	1.276	55.5	64.6

Supplementary Table 4. Current densities, Tafel slopes, and DL capacitances of doped $LaCoO_3$ thin films with conductive interlayer

		**Current density at 1.63 V vs. RHE [mA/cm^2]**	**Tafel slope [mV/dec]**	**DL Capacitance [μF/cm^2]**
**$LaCoO_3$**		1.49	61.6	58.2
**(Dopant) Al**	**5%**	1.09	61.0	61.7
	**10%**	0.84	60.5	61.0
	**20%**	0.38	57.0	57.0
**Sc**	**5%**	1.05	64.1	61.8
	**10%**	0.97	62.2	64.1
	**20%**	0.68	57.1	57.1
**Mn**	**5%**	1.11	57.5	69.5
	**10%**	0.91	57.6	68.6
	**20%**	0.53	55.2	58.1
**Fe**	**5%**	1.59	63.9	57.2
	**10%**	1.25	63.4	48.7
	**20%**	1.06	61.0	43.4
**Ni**	**5%**	1.12	55.4	63.2
	**10%**	0.83	51.8	66.2
	**20%**	0.80	55.5	61.8
**In**	**5%**	1.22	58.3	71.8
	**10%**	0.66	57.5	65.6
	**20%**	0.42	56.6	66.6
